# The ABCs of the atypical Fam20 secretory pathway kinases

**DOI:** 10.1016/j.jbc.2021.100267

**Published:** 2021-01-08

**Authors:** Carolyn A. Worby, Joshua E. Mayfield, Adam J. Pollak, Jack E. Dixon, Sourav Banerjee

**Affiliations:** 1 Department of Pharmacology, University of California, San Diego, La Jolla, California, USA; 2 Department of Cellular and Molecular Medicine, University of California, San Diego, La Jolla, California, USA; 3 Department of Chemistry and Biochemistry, University of California, San Diego, La Jolla, California, USA; 4 Division of Cellular Medicine, School of Medicine, University of Dundee, Dundee, UK

**Keywords:** extracellular matrix, Golgi, secretion, phosphorylation, signal transduction, enzyme structure, endoplasmic reticulum (ER), biomineralization, enzyme mutation, AI, amelogenesis imperfecta, DCM, dilated cardiomyopathy, DMP1, dentin matrix protein-1, EC, ectopic calcification, ER, endoplasmic reticulum, FGF-23, fibroblast growth factor-23, Fj, four-jointed, LDLR, low-density lipoprotein receptor, NC, nephrocalcinosis, PCSK9, proprotein convertase subtilisin-kexin, SIBLING, small integrin binding ligand-N-linked glycoprotein, T2D, type 2 diabetic, VLK, vertebrate lonesome kinase

## Abstract

The study of extracellular phosphorylation was initiated in late 19th century when the secreted milk protein, casein, and egg-yolk protein, phosvitin, were shown to be phosphorylated. However, it took more than a century to identify Fam20C, which phosphorylates both casein and phosvitin under physiological conditions. This kinase, along with its family members Fam20A and Fam20B, defined a new family with altered amino acid sequences highly atypical from the canonical 540 kinases comprising the kinome. Fam20B is a glycan kinase that phosphorylates xylose residues and triggers peptidoglycan biosynthesis, a role conserved from sponges to human. The protein kinase, Fam20C, conserved from nematodes to humans, phosphorylates well over 100 substrates in the secretory pathway with overall functions postulated to encompass endoplasmic reticulum homeostasis, nutrition, cardiac function, coagulation, and biomineralization. The preferred phosphorylation motif of Fam20C is SxE/pS, and structural studies revealed that related member Fam20A allosterically activates Fam20C by forming a heterodimeric/tetrameric complex. Fam20A, a pseudokinase, is observed only in vertebrates. Loss-of-function genetic alterations in the Fam20 family lead to human diseases such as amelogenesis imperfecta, nephrocalcinosis, lethal and nonlethal forms of Raine syndrome with major skeletal defects, and altered phosphate homeostasis. Together, these three members of the Fam20 family modulate a diverse network of secretory pathway components playing crucial roles in health and disease. The overarching theme of this review is to highlight the progress that has been made in the emerging field of extracellular phosphorylation and the key roles secretory pathway kinases play in an ever-expanding number of cellular processes.

The study of protein phosphorylation began as early as 1883 to 1900, when phosphorous was detected in milk casein (1) and egg-yolk phosvitin (2) respectively, thus making them the two earliest known phosphoproteins. Intriguingly, both these phosphoproteins are secreted from cells. Casein is secreted in milk (3) while phosvitin, a cleaved form of vitellogenin, is synthesized in the liver and secreted into the oviduct (4, 5). Since these initial discoveries, casein and phosvitin have been used as common artificial substrates in the study of numerous kinases (6, 7, 8). In fact, the first evidence for the existence of protein kinases was provided by the pioneering study of George Burnett and Eugene Kennedy where they used rat mitochondrial extract to provide ATP and casein as the substrate to demonstrate the covalent addition of phosphate to casein *in vitro* (6). Since that time, many investigators have added to the number and complexity of kinases leading to the compilation of the kinome in 2002 (9). This list of the human kinome included 540 individual members and represented kinases that could phosphorylate proteins as well as other biological molecules such as lipids and carbohydrates primarily within the cytosol and nucleus of the cell. But what about the kinases that phosphorylate resident proteins in the secretory pathway or proteins destined for secretion? This question was partially answered when the physiological secretory pathway kinase phosphorylating casein, family of sequence similarity 20C (Fam20C), was discovered in 2012 (10, 11). This same kinase was found to phosphorylate phosvitin in 2018 and thereby is accountable for the phosphorylation of the first identified secreted phosphoproteins (5).

The first clue for recognizing the secretory pathway kinases came from the identification of the *Drosophila* protein, four-jointed (Fj), as a secretory pathway kinase that phosphorylated the extracellular domains of atypical cadherins (12). Using Fj as a BLAST query revealed a small family of related proteins that included Fam20A, B, and C (11). Since little was known about these proteins, they were designated “Fams” based on shared but limited sequence similarity. They all harbor a signal peptide that would direct them into the secretory pathway, but due to a lack of sequence similarity with canonical kinases, none of these atypical kinases were represented in the human kinome. The other domain these proteins share, which is also the sequence of highest homology, is the C-terminal Fam20 domain. Unexpectedly, the conserved Fam20 domain in each of these proteins has a very different function. Fam20C is the Golgi casein kinase responsible for phosphorylating secreted proteins on SxE/pS motifs (11). Fam20A is a pseudokinase that interacts with Fam20C and increases its activity (13), and Fam20B is a xylose kinase involved in proteoglycan biosynthesis (14, 15).

Over the past few decades, multiple proteins in the extracellular and secretory space have been found to be phosphorylated. Many of these phospho-proteins are secreted into milk, serum, plasma, and cerebrospinal fluid (reviewed in (16)) and have defined roles in diverse cellular processes from signaling, coagulation, migration, extracellular matrix formation, proteolysis, and biomineralization. The majority of these secreted proteins exhibit a phospho-motif of SxE/pS but to date, we have limited knowledge of the function of the majority of these extracellular phosphorylation events (reviewed in (16)). Interestingly, out of the 540 kinases in the human kinome, only two kinases have been found localized in the secretory pathway: protein O-mannosyl kinase (POMK/SGK196) (17, 18) and the tyrosine kinase, vertebrate lonesome kinase (VLK/SGK493) (19), both of which do not phosphorylate SxE/pS motifs. Because the identity of the kinase(s) responsible for the majority of extracellular phosphorylation events remained elusive, the study of extracellular phosphorylation has lagged behind that of intracellular phosphorylation. It is increasingly clear that extracellular phosphorylation events play just as important roles in cellular regulation as their intracellular counterparts.

To date, there are 13 known secretory pathway kinases (or kinase-like proteins), and we know very little about some of them. In a handful of cases, we do not know their substrate specificity or even if they are active kinases. This review focuses on Fam20A, B, C, the small subfamily of secretory pathway kinases for which we have made significant progress. In particular, we will address their cellular functions, reported substrates, structure/function relationships, and importance in human disease.

## Secreted kinases

### VLK family and POMK

VLK and POMK are two secreted kinases that can be found at the root of the kinome tree. Therefore, their amino acid sequences were well enough conserved with the canonical kinases for them to be classified as kinases. POMK is an O-mannose kinase important for dystroglycan receptor function and matriglycan elongation (18, 20). VLK is the first secreted tyrosine kinase identified, and it phosphorylates a broad range of secreted and ER-resident substrates (19). A PSI-BLAST search using VLK as a query produces another small family of potential secreted kinases that includes Fam69A, Fam69B, Fam69C, DIA1, and DIA1R. Very little is known about these proteins (21, 22, 23).

### Fj family of atypical kinases

As alluded to in the introduction, the study of extracellular kinases was spearheaded by Ken Irvine’s laboratory when they published the first example of a secreted kinase, the fly protein Fj, which they went on to show phosphorylated unusual cadherin domains (12). The murine equivalent of Fj, four-jointed box 1 (FJX1) is involved in forming appropriate dendrite arbor morphology in the hippocampus (24), and recently, human FJX1 has been shown to increase the invasive potential of nasopharyngeal cancer cells (25, 26). In addition to FJX1 and Fam20A, B, and C, this small family contains two additional members, Fam198A and Fam198B. To date, neither Fam198A nor B has been ascribed kinase activity, and very little is known about their cellular functions (27, 28).

## Fam20B, the secreted xylose kinase

**Figure 1:**
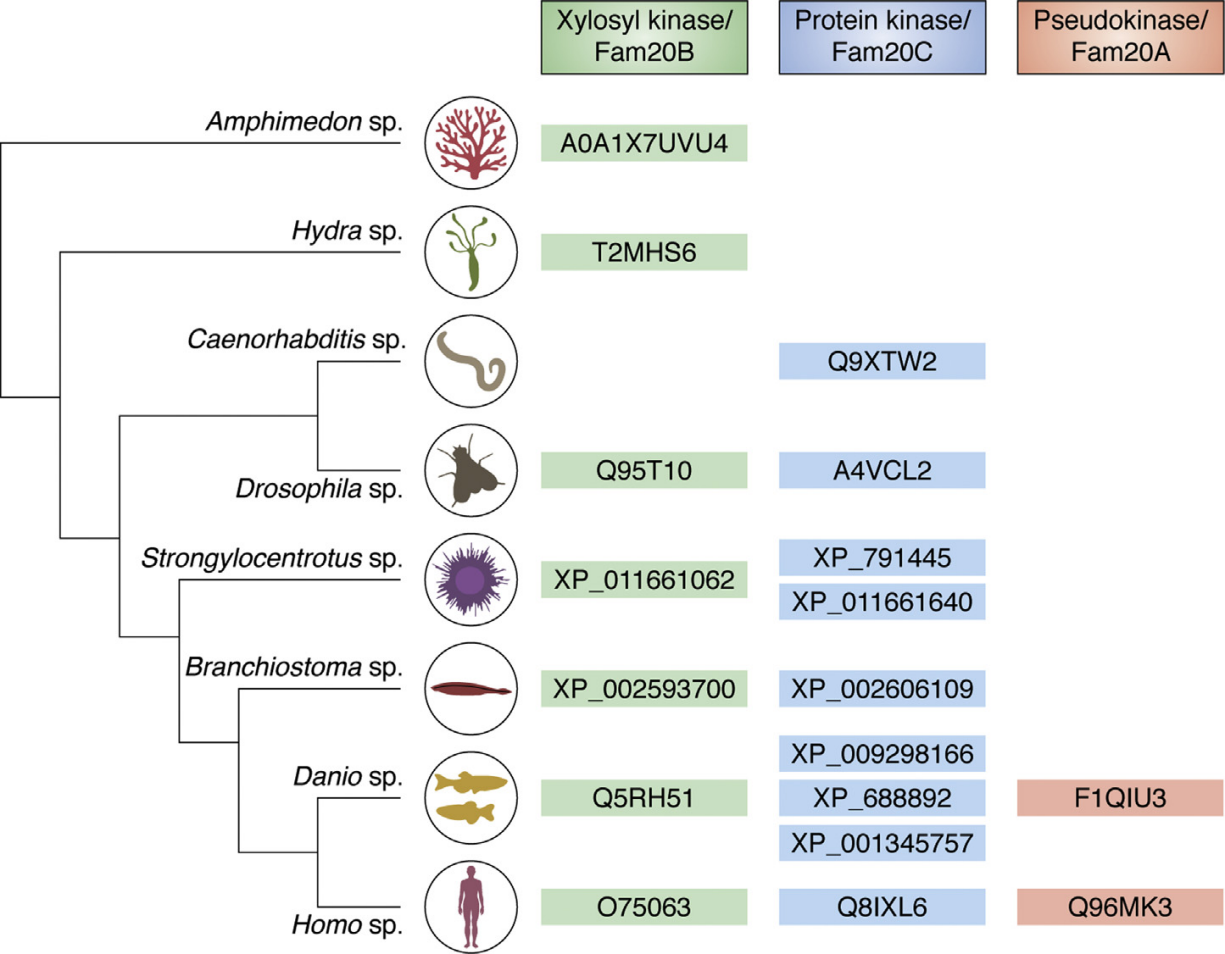
**Fam20 is conserved in animal kingdom.** Fam20B glycan kinase is the ancestral Fam20 kinase with a conserved role across the entire animal kingdom (nematode exception). Fam20C is first observed in nematodes with gene duplications observed in some organisms. In mammals only one copy of Fam20C is observed. Fam20A is observed in vertebrates only. (UniProt/UniParc accession IDs are provided).

**Figure 2:**
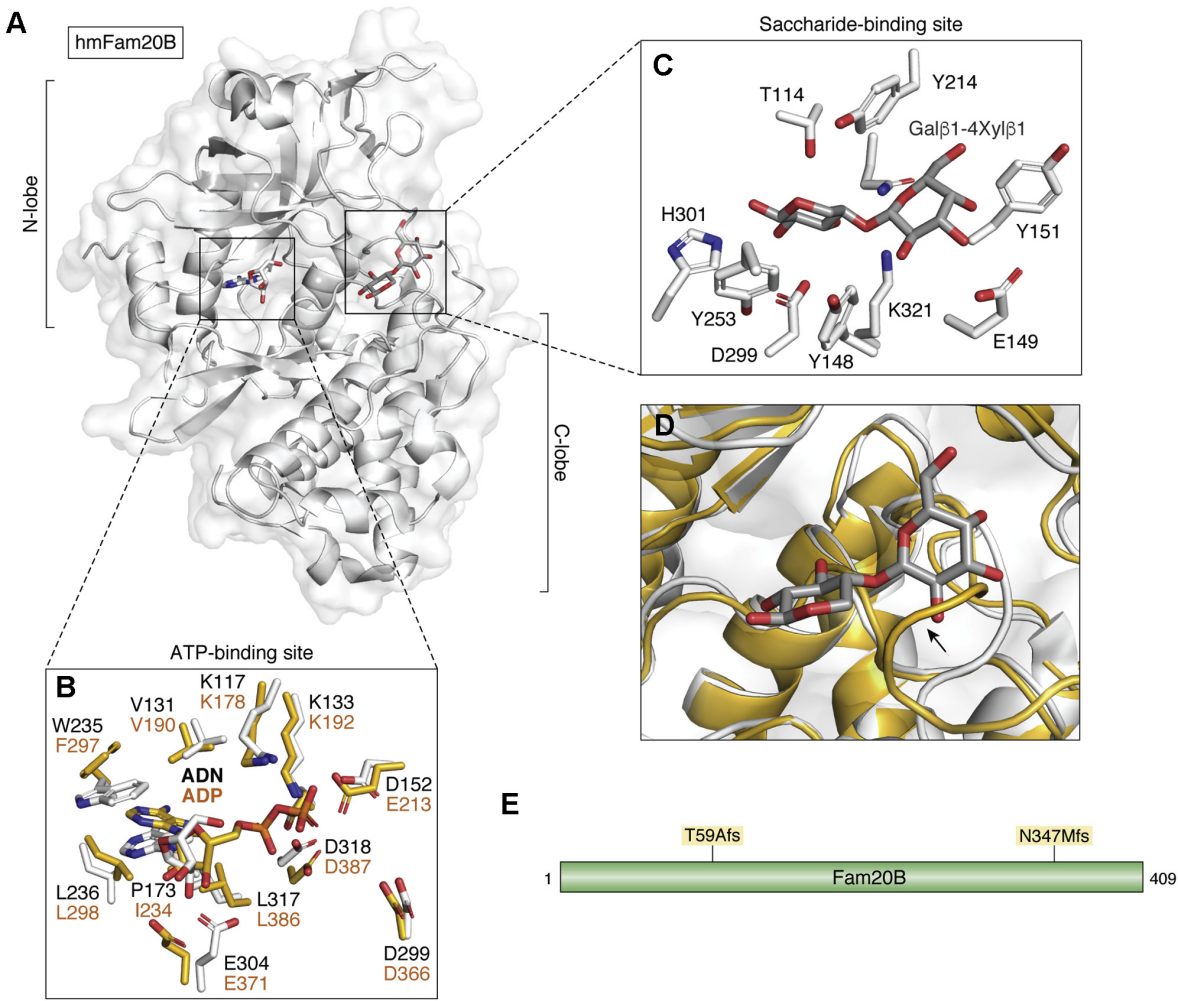
**Structure of FAM20B, the glycan kinase.***A*, structure of *Hydra magnipapillata* FAM20B (hmFAM20B, PDB ID: 5xoo, chain A, *white*) with bound adenosine (ADN) and Galβ1-4Xylβ1 substrate. N and C lobes indicated approximately. *B*, FAM20B ATP-binding site (PDB ID:5xoo, chain A, *white*, ADN:adenosine) is highly conserved with *C. elegans* FAM20C ATP-binding site (PDB ID:4kqb, chain A, *goldenrod*, ADP, adenosine diphosphate). Similar residues labeled (FAM20B:*black*, FAM20C:*orange*). *C*, FAM20B saccharide binding site containing Galβ1-4Xylβ1 substrate (*gray*) (PDB ID:5xoo, chain A). *D*, superimposed FAM20B (PDB ID:5xoo, chain A, *white*) with *C. elegans* FAM20C (PDB ID:4kqb, chain A) at saccharide binding site. *Arrow* indicates flexible loop occluding saccharide binding. *E*, gene diagram depicting disease mutations. fs, frame shift.

Vertebrates exhibit three members of the Fam20 family of proteins (Fam20A,B, and C), whereas early invertebrates such as hydra and sponge have a single homolog of Fam20 whose activity resembles the human Fam20B-like protein (Fig. 1) (29). Within the Fam20 family of secretory kinases, Fam20B was identified as a xylosylkinase kinase that phosphorylates xylose residues within the conserved tetrasaccharide linkages of proteoglycans (15). Interestingly, the xylose phosphorylation on the proteoglycan tetrasaccharide linkage was first identified in hydra (30), and further biochemical investigation revealed that hydra Fam20 and sponge Fam20 lacked protein kinase activity but exhibited robust xylosylkinase activity (29). In fact, Fam20B is thought to be the first ancestral template protein for the Fam20 family of kinases and the function of xylose phosphorylation is conserved through the animal phylum from sponges to humans (29). This evolutionary relationship is apparent in available structures. The ATP-binding sites of Fam20B and Fam20C are highly conserved (Fig. 2, *A* and *B*). However, Fam20B has a unique saccharide binding site not present in Fam20C or Fam20A (Fig. 2, *A* and *C*) (29). Fam20C homologs are characterized by an occluded substrate binding pocket that cannot accommodate bulky saccharide substrate due to steric clashes. This occlusion results from slight structural rearrangements arising from distal residue substitutions that position a flexible loop within the binding pocket (Fig. 2*D*) (29). The Fam20B-mediated xylose phosphorylation robustly stimulates galactosyltransferase II (GalT-II) activity leading to further addition of galactose to the tetrasaccharide linkages and accelerated proteoglycan chain extension (Fig. 5) (14). Furthermore, EXTL2 (Exostosin-Like Glycosyltransferase 2) polymerase utilizes the xylose phosphorylation to transfer a GlcNAc residue to the tetrasaccharide linkage region leading to termination of proteoglycan chain elongation (31). Intriguingly, depletion of Fam20B leads to immature proteoglycan formation, a phenotype quite reminiscent of Ehlers–Danlos syndrome, a rare inherited condition that affects connective tissue owing to GalT-II mutations (14). Thus, Fam20B plays an evolutionarily conserved quality-control role for proteoglycan biosynthesis and is arguably the ancestral Fam20.

Whole-body genetic depletion of Fam20B in mice was embryonic lethal at E13.5 with the embryos exhibiting severe development defects and significant organ hypoplasia (32). These observations were consistent with studies in zebrafish wherein loss-of-function mutants of Fam20B led to aberrant cartilage matrix organization and early stages of chondrocyte hypertrophy leading to skeletal defects (33). These initial *in vivo* observations were further echoed when tissue-specific depletion of Fam20B in mice led to the development of supernumerary teeth (34, 35), chondrosarcoma with major postnatal ossification defects (36), and severe craniofacial defects (37). Thus, the overarching role of Fam20B in proteoglycan biosynthesis likely contributes to the skeletal and developmental defects observed upon Fam20B depletion in tissue-specific *in vivo* models. In humans, two lethal compound heterozygous variants in Fam20B have been identified in a girl who died soon after birth (Fig. 2*E*) (38). The genetic alterations reported were T59Afs and N347Mfs and the patient exhibited severe organ hypoplasia, skeletal defects, and respiratory failure (38). The amino terminal T59A frameshift leads to hypomorphic gene function and essential loss of one allele of Fam20B. The carboxy-terminal alteration, N347M frameshift, results in disruption of more than 15% of the protein sequence and results in the loss of C389, which forms a disulfide bond with C332 and likely contributes to the global stability of the protein. The N347M frameshift, therefore, results in a destabilized Fam20B and also represents a functionally inactive variant. Intriguingly, osteoarthiritis and osteochondropathy patients with decreased proteoglycans and chondrocyte numbers exhibited marked reduction of Fam20B, GalT-II, and EXTL2 protein levels in knee cartilage biopsy samples (39). This suggests that Fam20B could be a predictive marker for specific bone diseases.

## Fam20C, the secreted Golgi casein kinase

**Figure 3:**
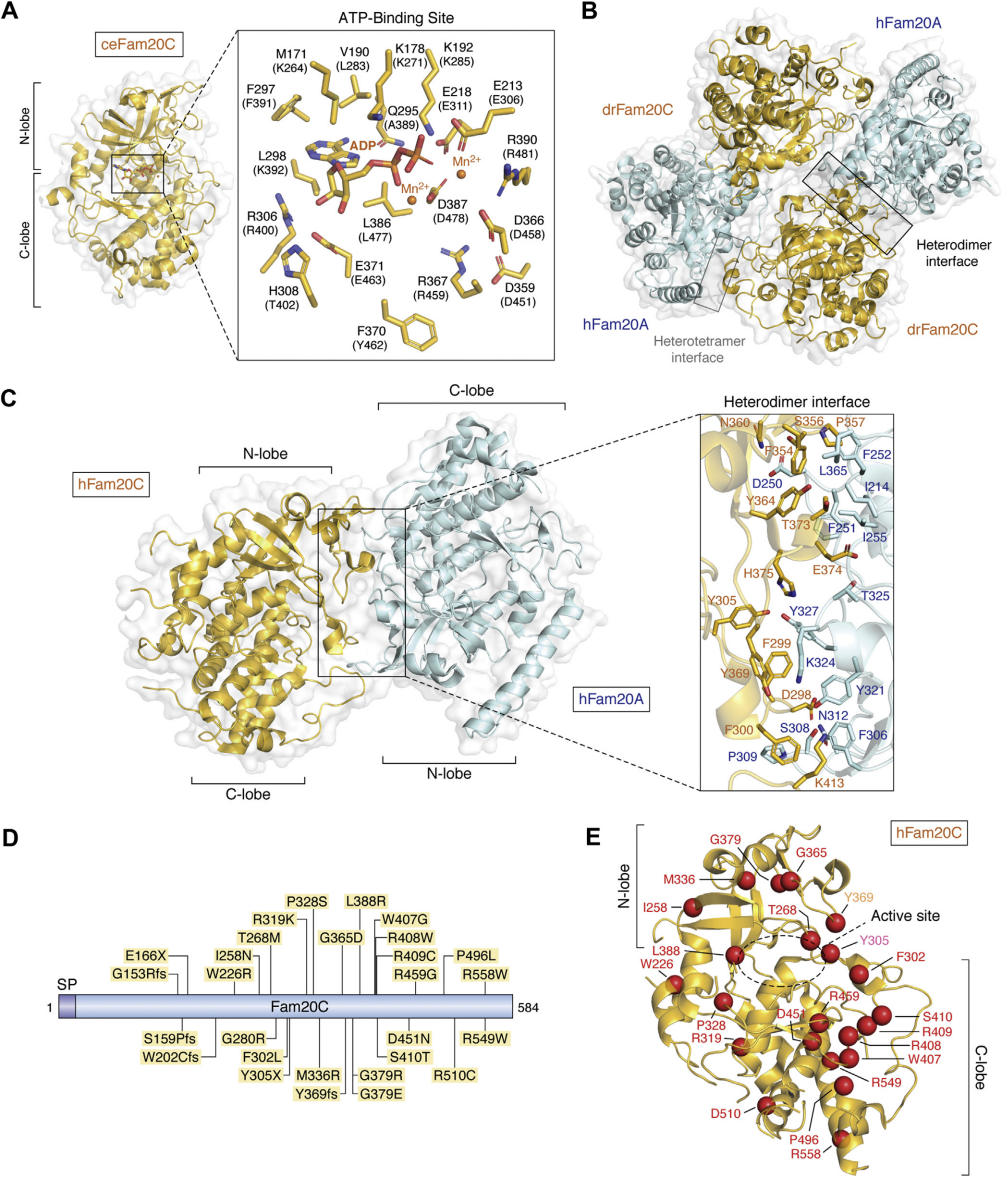
**Structure of Fam20C, the secreted protein kinase.***A*, structure of *C. elegans* FAM20C (ceFAM20C, PDB ID:4kqb, chain A, *goldenrod*). N and C lobe indicated approximately. ATP-binding site diagram of important residues. Parenthetical residues represent structurally equivalent residues in *Homo sapiens* FAM20C. *B*, heterotetramer of *Danio rerio* FAM20C (drFAM20C, *goldenrod*) and *Homo sapiens* FAM20A (hFAM20A, *cyan*) (PDB ID:5yh2; chains A–D). Heterodimer interface and heterotetramer interfaces indicated. *C*, heterodimer of *Homo sapiens* Fam20C (hFAM20C, goldenrod1) and *Homo sapiens* FAM20A (hFAM20A, *cyan*) (PDB ID:5yh3, chains A and C). Residues important to the heterodimer interface indicated. N and C lobe indicated approximately. *D*, gene diagram depicting disease mutations (fs, frame shift; X, STOP/termination). *E*, cartoon depiction of kinase indicated positions of mutated residues when resolved (mutations as *red spheres*, PDB ID:5yh3, chain C). Residue labels color coded to indicate mutation type (*red*: missense mutation, *orange*: frameshift, and *pink*: STOP/termination). N and C lobes indicated approximately.

As stated previously, the story of milk casein as a phosphoprotein started in the late 19th century when Olof Hammarsten reported the presence of phosphorus in casein (1). Fifty years later, Fritz Lipmann identified that the phosphorus was covalently bound to casein as phosphoseryl groups (40). Eventually, the sequences surrounding those phosphoseryl groups in casein were identified as SxE/pS, which prompted the idea that SxE/pS sequence was the preferential motif for enzymes phosphorylating casein (41, 42) within the secretory pathway (43). In subsequent years, two cytoplasmic kinases were shown to robustly phosphorylate casein *in vitro* and because of this ability were designated casein kinase 1 and 2 (44). This was despite the fact that they would never come into contact with casein because they were localized to the cytoplasm and nucleus while casein, a secreted protein, resided in the secretory pathway and extracellularly. The bona fide “Golgi casein kinase” activity was initially observed in lactating mammary glands (41, 43, 45) and partially purified from milk (46). Lorenzo Pinna and colleagues extensively characterized the activity of the partially purified protein from Golgi fractions and further reported that the kinase was highly resistant to the majority of the well-established kinase inhibitors including staurosporine (3, 47, 48, 49, 50). In 2012, this elusive activity was identified molecularly when Fam20C was experimentally recognized to be the Golgi casein kinase capable of phosphorylating casein *in vivo* (11). Although atypical, crystallography studies on the nematode-ortholog of Fam20C (51) revealed that the kinase exhibited the canonical N- and C-lobed kinase structure with a well-defined ATP-binding active-site pocket (Fig. 3*A*). The breadth of Fam20C’s activity was alluded to when phosphoproteomic studies of human plasma, serum, and cerebrospinal fluid demonstrated that more than two-thirds of secreted phosphorylated proteins were phosphorylated on SxE/pS motifs (52, 53, 54). In fact, phosphoproteomic analysis of secreted neuropeptides in the nervous and endocrine system revealed that the predominant phospho-motif was SxE (55). This was solidified by studies in which Fam20C was ablated in several tissue culture cell lines and the culture media was analyzed for secreted phosphoproteins (56). Cumulatively, this work resulted in affirming that Fam20C is the kinase responsible for phosphorylating the majority of secreted proteins and broadened Fam20C’s substrate preference to include phosphorylation sites other than SxE/pS sites (56). For instance, a recent study reported that specific threonine residues on the neuroendocrine chaperone 7B2 were phosphorylated by Fam20C (57). Surprisingly, there were nonoverlapping substrates between the secreted phosphoproteome from the different cell lines indicating that individual cell populations have different milieux of secreted proteins.

Fam20C has a strong cofactor preference for Mn^2+^ and Co^2+^ ions over the canonical Mg^2+^ ion for its kinase activity (47) although the physiological levels of Mg^2+^ in cells (around 1 mM) are 10^4^ fold higher than Mn^2+^ (about 100 nM) (44). Lorenzo Pinna and colleagues argued that under physiological circumstances, specific signaling components may play a role in promoting Fam20C to utilize Mg^2+^ over Mn^2+^ in the secretory pathway (44). The group reported that sphingosine and sphingosine-1-phosphate significantly improved the ability of Fam20C to utilize Mg^2+^ as a cofactor (50, 58). Indeed, sphingosine addition led to an eightfold higher activity of Fam20C *in vitro* with a threefold increase in Vmax and a consequent threefold decrease in Km (50, 58). However, ceramide, the precursor of sphingosine, had no effect on Fam20C activity, thus suggesting sphingosine as a specific activator of Fam20C (50, 58). Interestingly, the activity of Fam20C is dynamically controlled by its binding partner Fam20A (Fig. 3, *B* and *C*). Fam20A and Fam20C together form a heterodimeric complex (Fig. 3*C*), which dramatically promotes the activity of Fam20C to phosphorylate its substrates (13, 29). Two heterodimers can further associate to form a heterotetrameric complex (Fig. 3*B*), but it remains an open question as to which form exists *in vivo*. This uncommon allosteric mode of pseudokinase-mediated activation of Fam20C is further explained below in the Fam20A section. Finally, functional annotations of Fam20C substrates suggest that Fam20C will play important roles in many physiological processes and disease states.

### FAM20C substrates in nutrition and mineralization

The gene encoding casein resides on chromosome 4 surrounded by other genes encoding proteins that contain multiple SxE motifs. Casein accounts for approximately 80% of the total protein in bovine milk where it interacts with calcium phosphate forming colloidal structures called casein micelles, thereby providing nutrients including calcium and phosphate for growth of bones and teeth to mammalian infants (59). The consequences of casein phosphorylation have been intensively studied with regard to cheese manufacturing where it is suggested to affect milk technological properties by stabilizing calcium phosphate nanoclusters and promoting micellar growth (59, 60, 61).

In addition, chromosome 4 harbors another gene cluster encoding the small integrin binding ligand-N-linked glycoproteins (SIBLINGs). These genes are known to regulate bone and tooth development and encode osteopontin, dentin matrix protein-1 (DMP1), matrix extracellular phosphoglycoprotein, bone sialoprotein, and dentin sialophosphoprotein, all of which are involved in binding calcium and all of which are Fam20C substrates (11). In fact, Fam20C phosphorylates DMP1 in osteoblasts and young osteoclasts, which leads to the secretion of phospho-DMP1 into the pericanalicular matrix of mineralized bone (62). Fam20C is further thought to indirectly promote DMP1 transcription (63). In addition, Fam20C phosphorylates multiple sites on osteopontin and promotes its secretion (64) but inhibits its binding to αvβ3 integrin (65). These negatively charged phosphorylated substrates allude to Fam20C’s involvement in Ca^2+^ regulation in many varied and diverse processes including nutrition and the formation of mineralized tissues. Indeed, a large body of literature, focusing on conditional tissue-specific knockout mice and cell models, reports the roles of Fam20C in promoting biomineralization including the growth and development of osteoblasts, osteoclasts, bone, dentin, and enamel (Fig. 5) (66, 67, 68, 69, 70, 71, 72, 73, 74, 75, 76, 77, 78).

### Fam20C substrates promoting secretion and ER homeostasis

Phosphoproteomic analysis of pancreatic β -islet cells from type 2 diabetic obese (T2D) mice revealed 39 potential phosphosites conforming to the SxE motif (79). The study reported that Fam20C levels went up in the cells of T2D mice, thereby promoting secretion of immature proinsulin under hyperglycaemic conditions (79). Upon restoring euglycaemia, the levels of Fam20C and 11 corresponding SxE phosphosites were brought back to basal level (79). This study suggests that Fam20C might play an important role in the control of insulin section from the β -islet cells of pancreas. In fact, recent studies suggest Fam20C plays a pivotal role in ER homeostasis, which promotes proper section, including phosphorylation of proteins sequestered within the secretory pathway (Fig. 5). Recent works report that Fam20C phosphorylation of ER oxidoreductin 1α (Ero1α) on Ser145 (SxE site) is important for regulating ER redox homeostasis and oxidative protein folding (80). This Ero1α phosphorylation is induced following secretion-demanding conditions such as lactation and interestingly, this posttranslational event occurs in the Golgi apparatus, and Ero1α is retrograde-transported to the ER mediated by ERp44 (80). Furthermore, Fam20C maintains ER proteostasis and protects against ER stress-induced cell death (81). Protein disulfide isomerase (PDI) is a highly abundant ER-resident enzyme playing critical roles as both a thiol-disulfide oxidoreductase and a molecular chaperone, which prevents protein misfolding in the ER (82, 83). Fam20C phosphorylates PDI on Ser357 upon ER stress and promotes the activity of PDI to maintain ER proteostasis (81). Indeed, loss of Ser357 (Ser359 in mouse) leads to acute liver damage in mice challenged with proteotoxic stress (81). Interestingly, recent studies show that Fam20C phosphorylation is required for the secretion of certain proteins. For example, Fam20C phosphorylates calcium binding protein 45 kDa (Cab45), a Golgi protein, regulating the sorting and secretion of proteins (84). This phosphorylation regulates Cab45 oligomerization independent of its Ca^2+^ binding ability and facilitates translocation of Cab45 into trans Golgi network-derived vesicles, thus accelerating vesicle budding (84). Furthermore, the Cab45 phosphorylation enhances secretion of its client proteins, including lysozyme C (84). Similarly, Fam20C phosphorylation has been shown to be important for the secretion of osteopontin (64).

### Fam20C substrates in blood

Phosphoproteomic analyses of plasma and serum revealed that the majority of phosphorylated sites identified adhered to the SxE/pS motif (52, 54), thus triggering the hypothesis that the majority of the extracellular plasma/serum phosphoproteins could be Fam20C substrates. Multiple proteins with well-established roles in blood coagulation, phosphate homeostasis, and complement pathways have been identified in phosphoproteomic studies by comparing the phosphoproteome of wild-type cells with cells lacking Fam20C (56, 85) (Fig. 5). The major vertebrate clotting factor fibrinogen (alpha and gamma chains) was identified as a potential substrate of Fam20C in these phosphoproteomic screens (56). Phosphorus was found in fibrinogen as early as 1962 and the amino acid sequence revealed the sites to be SxE (86). During tissue and vascular injury, fibrinogen is cleaved by thrombin to fibrin peptides, which form a fibrin-based blood clot and stop bleeding (87). It has been reported that phosphorylated fibrinogen binds better to thrombin, thus releasing more fibrin peptides and promoting faster coagulation (88, 89). Fam20C has been found to directly phosphorylate fibrinogen alpha and gamma chains *in vitro* (56), and further work is needed to define the physiological roles of the phosphorylation events. On a similar note, Fam20C phosphorylates the A2 domain of von Willebrand factor (vWF) on two SxE sites, pSer1517 and pSer1613 (90). The modifications promote platelet adhesion to sites of vascular injury and helps in coagulation (90). Among the other serum/plasma proteins identified as Fam20C substrates are collagen and the complement components C3 and C4 (56) wherein collagen and C3 have been reported to be phosphorylated previously (91, 92). Further work is needed to establish the role of Fam20C and phosphorylation of its key substrates in the blood coagulation pathway.

Another well-characterized substrate of Fam20C in serum is fibroblast growth factor-23 (FGF23), a bone-derived hormone that regulates serum phosphate levels (85, 93). Mice with Fam20C deletion exhibit an increase in bioactive serum FGF23 leading to the development of hypophosphatemic rickets and skeletal defects (32, 76), which can be partially reversed by feeding the mice a high-phosphate-containing diet (94). In fact, within the Golgi, Fam20C phosphorylates FGF23 on Ser180 (SxE site), which inhibits its O-glycosylation and subsequently promotes proteolysis and inactivation of the hormone (85). Intriguingly, proteolysis-resistant missense alterations adjacent to Ser180 (R176Q, R179W, and R179Q) activate FGF23 leading to hypophosphatemic rickets (95). Furthermore, knockdown of Fam20C in cells promotes FGF23 mRNA expression (63), and elevated levels of serum FGF23 contribute to cardiovascular complications and increased mortality in patients with chronic kidney disease (96).

### Fam20C substrates in heart

Besides FGF23, which contributes directly to cardiovascular problems in patients, various other substrates of Fam20C have been implicated in heart disease (Fig. 5). PCSK9 (proprotein convertase subtilisin-kexin 9) patient genetic variations altering SxE sites correlate with LDL-cholesterol dysregulation, a risk factor for heart disease (97). Importantly, Fam20C-mediated phosphorylation of PCSK9 improves PCSK9 secretion and enhances the degradation of the low-density lipoprotein receptor (LDLR) in endosomes/lysosomes (97). On a similar note, PCSK7 is phosphorylated by Fam20C on Ser505 (SxE site) leading to higher triglyceride uptake into adipocytes (98). Interestingly, exome sequencing revealed a low frequency coding variant PCSK7, R504H, correlated with 30% lower plasma triglyceride levels in individuals harboring this change (98). Further biochemical analyses revealed that the R504H substitution enhanced phosphorylation of the adjacent S505 possibly promoting higher triglyceride uptake (98).

Cardiac function, contraction and relaxation, is brought about by a complex interplay of multiple proteins and posttranslational modifications playing essential roles in regulating intracellular calcium (Ca^2+^) handling (99). The sarcoplasmic reticulum (SR) of cardiac muscle is the Ca^2+^ storage organelle, and Ca^2+^ is shuttled between the SR and cytosol *via* various SR resident receptors during contractions and relaxations of the heart (100). Fam20C resides in the SR of cardiac muscle and phosphorylates multiple major Ca^2+^ handling machinery proteins including histidine-rich Ca-binding protein (HRC), Stim1, calsequestrin 2, sarcalumenin, triadin, calumenin, and calreticulin (101, 102). These proteins play essential roles mediating SR Ca^2+^ storage, uptake, and release (102, 103). For example, Fam20C-mediated phosphorylation of calsequestrin 2, the major Ca^2+^ binding protein in the SR, dramatically alters the ability of calsequestrin 2 to oligomerize, which is critical to its function (102). Stim1, the luminal ER/SR Ca^2+^ sensor responsible for store-operated Ca^2+^ entry in a variety of cell types, is also dramatically regulated by Fam20C phosphorylation, providing the most compelling evidence of Fam20C-mediated Ca^2+^ regulation. In addition, a recently discovered Stim1-S88G substitution (within an SxE site) was found in a patient with heart disease and the substitution, which precludes Fam20C phosphorylation, was shown to alter Ca^2+^ signaling (102, 104).

Interestingly, cardiomyocyte-specific Fam20C knockout mice (cKO) exhibited signs of heart failure upon aging or induced pressure overload by transverse aortic constriction (102). At 9 months of age, cKO mice exhibited a significant increase in left ventricle chamber size with distinct features of heart fibrosis and dilated cardiomyopathy (102). The heart failure phenotype in cKO mice is thought to be brought about by dramatic SR Ca^2+^ handling defects since isolated cardiomyocytes from aged cKO mice exhibited severe Ca^2+^ cycling defects and delayed relaxation (102).

Dilated cardiomyopathy (DCM) is an underlying heart defect and is associated with sudden death in over 50% of the cases (105). Aged cKO mice exhibit clear signs of DCM, and although multiple substrates have been reported for Fam20C in SR, HRC has been widely implicated in DCM (103). HRC is an essential Ca^2+^ handling protein, and its depletion leads to enhanced cardiomyocyte aftercontractions upon stress (106). Failing human hearts exhibit lower protein levels of HRC, and multiple genetic variants of HRC have been reported in human DCM cases (103). Fam20C-mediated phosphorylation of HRC is thought to control Ca^2+^ leak and enhance SR Ca^2+^ transport, thereby maintaining ambient signaling (101). The site of phosphorylation on human HRC is S96, which is a canonical SxE phosphorylation site (101). Remarkably, S96A is a common human genetic variant of HRC, and patients with the homozygous Ala/Ala variant exhibit fourfold increased risk of lethal ventricular arrhythmias in idiopathic DCM compared with normal Ser/Ser patients and twofold increased risk when compared with heterozygous individuals (103). Furthermore, preliminary genetic analysis indicates that roughly 60% of participants had at least one copy of S96A suggesting that this condition has extremely broad implications for heart disease (103). The intriguing dosage-dependent manner of DCM lethality in the nonphosphorylatable S96A genetic variant of HRC suggests that pS96 HRC phosphorylation by Fam20C is likely an important molecular event in cardioprotection.

### Fam20C genetic alterations in disease

Biallelic loss-of-function genetic alterations in the Fam20C gene lead to the development of an autosomal recessive disorder called Raine syndrome (OMIM #259775) Figure 3*D* (107, 108, 109). In 1985, two infant sisters with neonatal lethality were reported to exhibit a unique, autosomal recessive case of congenital sclerosing osteomalacia with cerebral calcification (110). It was not until 2016 that their archival DNA was sequenced to reveal a Fam20C genetic alteration in a key conserved region (111). These patients may have been arguably the first documented cases of Raine syndrome harboring genetic alterations in Fam20C. The name “Raine syndrome” was coined in 1989 when Raine and colleagues comprehensively reported this lethal osteosclerotic bone dysplasia (112) while links with Fam20C alterations were established by Simpson and colleagues in 2007 (107). The cases presented often exhibit neonatal-lethality with extreme skeletal deformities, ectopic calcification, and organ hypoplasia (107). Some nonlethal cases have also been reported with patients exhibiting hypophosphatemia, altered facial and skeletal features (108). Over 40 cases of Raine syndrome have been reported worldwide and DNA sequencing revealed that all these patients carried various alterations in the Fam20C gene, which are likely the driving cause of disease (107, 108, 109). About 25 unique alterations have been reported for Fam20C in disease, which affect stability, secretion, activity, and integrity of Fam20C protein (Fig. 3*D*) (11, 51). Intriguingly, a direct correlation has been observed between Fam20C activity and disease lethality, wherein, complete deletion leads to neonatal lethality, whereas residual activity is sufficient to keep the individual alive beyond birth to preteen and even teenage years. Two teenagers with hypophosphatemia and rickets exhibited a compound heterozygous Fam20C genetic alteration where one copy of the Fam20C gene contained a T268M substitution (113). Fam20C T268M purified *in vitro* preserved only 10% of wild-type kinase activity (50). Interestingly, FDA-approved multiple sclerosis drug and sphingosine analog, fingolimod, potently activated Fam20C *in vitro* (50). Fingolimod also led to higher activity of Fam20C T268M *in vitro* (50). This suggests that fingolimod may be utilized in partially alleviating the loss of activity of Fam20C in nonlethal Raine syndrome patient cases. Furthermore, a similar amino acid replacement Ser to Thr (S410T) in a patient exhibited very mild symptoms (114). In fact, a canine model of nonlethal Raine syndrome has been reported exhibiting a minimally disruptive Ala to Val substitution in the Fam20C kinase domain (115). Most alterations reported alter the protein sequence of Fam20C in key conserved regions, whereas large chromosomal rearrangements (107) and splice-site alterations also result in Fam20C deletions and disease manifestations (116, 117). The reported Fam20C disease alterations in humans with the exception of splice-site mutations have been listed in Table 1 and Figure 3, *D* and *E* with corresponding information on inheritance, lethality, and effect on Fam20C protein/kinase activity.

**Table 1:** Fam20C genetic alterations in human disease

Amino acid	Inheritance	Disease	Possible effect	Reference
G153Rfs	CH	Lethal, Raine syndrome	Loss of kinase domain, alters 74% of protein sequence	(140)
S159Pfs	CH	Lethal, Raine syndrome	Loss of kinase domain, alters 73% of protein sequence	(140)
E166X	Homo	Lethal, Raine syndrome	Loss of kinase domain, removes 72% of protein sequence	(141)
W202Cfs	Homo	Nonlethal, mild Raine syndrome	Disruption of kinase domain, alters 65% of protein sequence	(116)
W226R	Homo	Nonlethal, Amelogenesis imperfecta	Unknown, Possibly important for protein folding	(141, 142)
I258N	CH	Nonlethal, severe skeletal deformities	Affects Fam20C folding and secretion	(11, 51, 108)
T268M	CH	Nonlethal, mild Raine syndrome, hypophosphatemia	90% loss of kinase activity and reduced secretion	(50, 51, 113)
G280R	CH	Nonlethal, severe skeletal deformities	Affects Fam20C folding and secretion	(11, 51, 108)
F302L	CH	Nonlethal, hypophosphatemic osteomalacia with osteosclerosis	Unknown, possibly important for protein folding and/or Fam20A/C tetramer formation	(143)
Y305X	CH	Nonlethal, mild Raine syndrome, hypophosphatemia	Hypomorphic, removes 48% of protein sequence.	(113)
R319K	CH	Lethal, multiple defects including Raine syndrome	Unknown	(144)
P328S	Homo	Lethal, Raine Syndrome; Nonlethal in two siblings, severe retardation and skeletal abnormalities	Affects Fam20C folding and secretion	(11, 51, 109)
M336R	Homo	Lethal, Raine syndrome	Affects N-glycosylation, protein folding, and secretion of Fam20C	(145)
G365D	Het	Lethal, Raine syndrome	Possible loss of function of Fam20C	(111)
Y369fs	CH	Nonlethal, mild Raine syndrome	Hypomorphic, alters 37% of protein sequence	(146)
G379R	Homo	Lethal, Raine Syndrome	Affects Fam20C folding and secretion	(11, 51, 108)
G379E	CH	Lethal, Raine Syndrome	Affects Fam20C folding and secretion	(11, 51, 108)
L388R	Homo	Lethal, Raine Syndrome	Affects Fam20C folding and secretion	(11, 51, 107)
W407G	Homo	Nonlethal, craniofacial anomalies, intracranial calcification, developmental delay	Unknown, Possibly important for protein folding	(147)
R408W	Homo	Nonlethal, mild Raine Syndrome hypophosphatemia	Diminishes Fam20C activity to 50%	(51, 148)
R409C	Homo	Lethal, Raine syndrome	Unknown	(149)
S410T	Homo	Nonlethal, mild skeletal issues	Minimal structural disruption expected	(114)
D451N	Homo	Lethal at preteen, Raine Syndrome. Nonlethal cases reported	Disrupt salt-bridge in catalytic segment and affects Fam20C secretion	(11, 51, 108, 141)
R459G	CH	Nonlethal, mild Raine syndrome	Mutation adjacent to cation interacting Asp	(146)
P496L	Homo	Nonlethal, mild Raine symptoms	Likely disruption of Fam20C activation loop	(116)
R510C	CH	Lethal, multiple defect, Raine syndrome	Unknown	(144)
R549W	Homo/CH	Lethal/Nonlethal, Raine Syndrome	Affects Fam20C folding and secretion	(11, 51, 107, 117)
R558W	Homo	Lethal, Raine Syndrome	Unknown	(150)
45, XY (7;7) (p22;p22)	CH	Lethal, Raine syndrome	Microdeletion	(107)
46, XY[hg19] 7p22.3 (36480–523731)	Homo	Lethal, Raine syndrome	487 kb deletion including *FAM20C*	(151)

CH, Compound heterozygous; fs, Frameshift; Het, Heterozygous; Homo, Homozygous; X, STOP/Termination.

Clinical presentation is heterogeneous and the classifications presented here reflect the symptomology reported in the literature.

## Fam20A, the secreted pseudokinase

**Figure 4:**
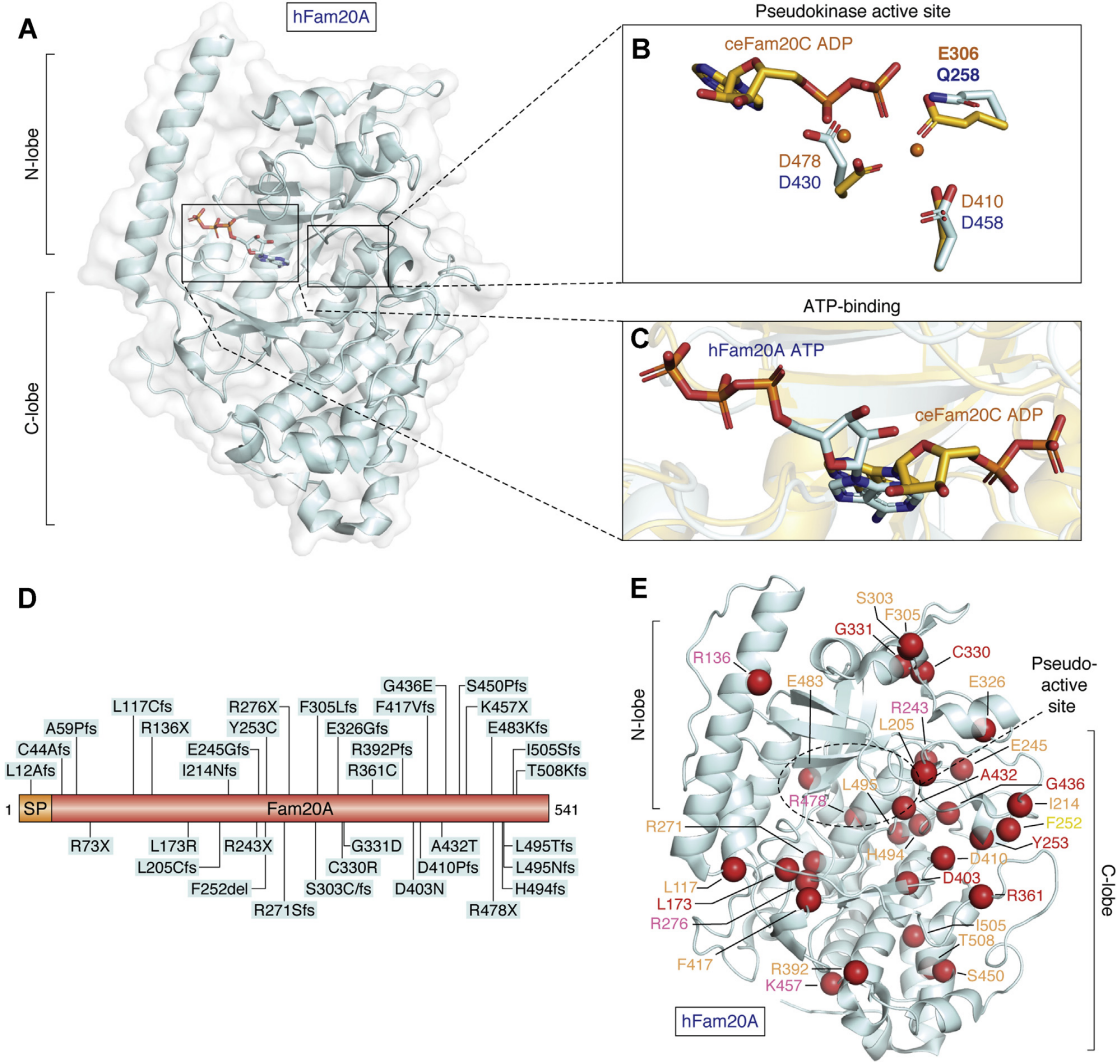
**Structure of Fam20A, the secreted pseudokinase.***A*, structure of *Homo sapiens* FAM20A (hFAM20A, PDB ID:5yh3, chain A, *cyan*). *Boxes* indicate the pseudokinase active site and ATP-binding site. N and C lobes indicated approximately. *B*, superimposition of *Homo* sapiens FAM20A (hFAM20A, PDB ID:5yh3, chain A, *cyan*) and *C. elegans* FAM20C (ceFAM20C, PDB ID:4kqb, chain A, *goldenrod*) active sites. Manganese coordinating residues indicated. Q258 abolishes manganese and ATP-binding. ceFAM20C ATP-binding displayed for reference. *C*, superimposition of *Homo* sapiens FAM20A (hFAM20A, PDB ID:5yh3, chain A, *cyan*) and *C. elegans* FAM20C (ceFAM20C, PDB ID:4kqb, chain A, *goldenrod*) bound ATP/adenosine diphosphate (ADP). hFAM20A binds ATP in an inverted fashion. *D*, gene diagram depicting disease mutations (del, deletion; fs, frame shift; X, STOP/termination). *E*, cartoon depiction of kinase indicated positions of mutated residues when resolved (mutations as *red spheres*, PDBID:5yh3, chain C). Residue labels color coded to indicate mutation type (*red*: missense mutation, *orange*: frameshift, *pink*: STOP/termination, and *yellow*: deletion). N and C lobes indicated approximately.

**Figure 5:**
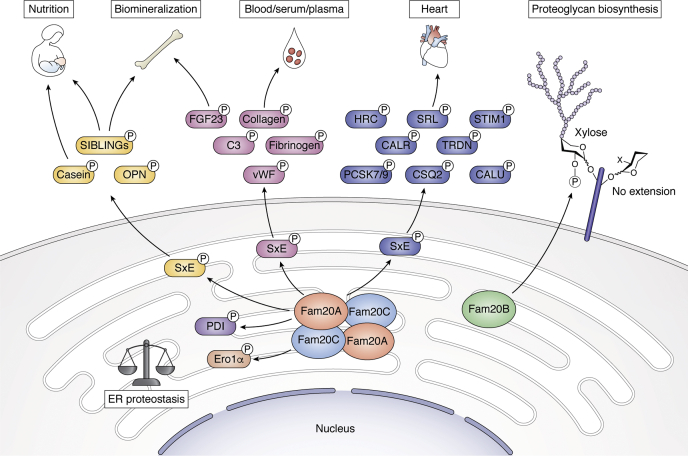
**Roles of Fam20 secretory pathway kinases.** The overarching roles of the Fam20 kinases identified to date are in nutrition, biomineralization, blood, cardiac function, proteoglycan biosynthesis, allosteric kinase activation, and endoplasmic reticulum proteostasis. Fam20 paralogs are localized in the secretory pathway and phosphorylate multiple substrates playing essential roles in animal physiology.

Unlike Fam20C, which is ubiquitously present in all tissues, Fam20A is preferentially expressed in lactating mammary glands and in enamel and dental matrices (13, 32). Fam20A forms a functional heterotetrametric complex with Fam20C (Fig. 5) and allosterically increases Fam20C activity, *via* heterodimerization, toward its substrates (Fig. 3, *B* and *C*) (13, 29). Interestingly, formation of the heterodimer is sufficient to allosterically increase Fam20C activity both *in vitro* and in cells, and the unique contributions of the heterotetramer are still unknown (29). Fam20A is a paralog of Fam20C and is the first secreted pseudokinase identified (Fig. 4, *A* and *B*) (13). Pseudokinases are proteins that share sequence homology with kinases but lack kinase activity either due to mutations in normally conserved amino acids that catalyze phosphoryl transfer (118) or utilize the kinase fold to transfer molecules other than phosphate (119, 120). A conserved Gln residue in Fam20A replaces a Mn^2+^ cation coordinating Glu residue of Fam20C, which is essential for catalysis (13). In fact, mutagenesis studies revealed that replacing the Gln to a Glu in Fam20A triggered hydrolysis of ATP and restored kinase activity (13). In addition to the lack of an essential residue required for catalysis, Fam20A binds to ATP (Fig. 4, *A* and *C*) in a unique conformation (121). Structural studies revealed that the ribose moiety of the ATP is “upside down,” and the entire nucleotide is inverted with the phosphate groups pointing at the opposite direction (121). Hence, the γ-phosphate is positioned away from the active site and cannot be utilized for transfer. Several hydrophobic residues and hydrogen bonds in the pseudokinase pocket bind the adenine of ATP (Fig. 4, *B* and *C*) while the otherwise-hydrolyzable γ-phosphate is surrounded and stabilized by extensive salt bridge and hydrogen bonds (121). Furthermore, the “inverted” ATP-binding to Fam20A seems to prefer the absence of metal ions as biochemical studies indicated that the dissociation constant of Fam20A ATP-binding is 50-fold higher in the presence of Mn^2+^ cation (121). Intriguingly, ion-independent ATP-binding of Fam20A remarkably promoted the formation and structural homogeneity of the heterotetrameric Fam20A-Fam20C complex (121). Although cation-independent ATP-binding has been reported previously in other pseudokinases (118, 122, 123), the inverted binding to ATP and the heterotetramer formation in the secretory pathway make Fam20A a unique pseudokinase. Interestingly, subtle structural differences from Fam20C redesign Fam20A’s ability to achieve kinase-independent function (121). Fam20A has a unique and highly conserved insertion in the Gly-rich loop, which triggers the formation of two unique disulfide bonds (human Fam20A: Cys209-Cys319 and Cys211-Cys323) (121), and truncation of this insertion due to aberrant RNA splicing leads to the development of tooth enamel defects called amelogenesis imperfecta in a patient (124).

Variations in the gene encoding Fam20A result in amelogenesis imperfecta (AI), nephrocalcinosis (NC), and ectopic calcification (EC) (125). Similar observations were echoed from whole-body and tissue-specific genetic depletion of Fam20A in mice, which exhibited clear phenotypes of AI and dental defects (32, 126). An exhaustive list of Fam20A patient variations with corresponding clinical information has been reported by Nitayavardhana and colleagues in 2020 (127). To date, about 40 different disease-causing genetic alterations have been reported in Fam20A in 70 patients of 50 independent families (Fig. 4*D*) (127). The patients exhibited nonlethal dental symptoms including hypoplastic enamel, gingival hyperplasia, and unerupted permanent teeth (127). The majority of the alterations were frameshifts with increased chances of hypomorphism, truncation, deletion, complete loss of function, major structural effects with possible dissociation from the Fam20A–Fam20C complex. The alterations are listed in Table 2 and Figure 4, *D* and *E*.

**Table 2:** Fam20A genetic alterations in human disease

Amino acid	Inheritance	Disease	Possible effect	Ref
L12Afs	Homo; CH	AI	Deletion/Hypomorphic	(124, 152, 153, 154)
C44Afs	CH	AI, NC	Deletion/Hypomorphic	(155)
A59Pfs	Homo	AI	29 bp duplication/Hypomorphic	(156)
R73X	CH	AI, NC	Deletion/Hypomorphic, removes 87% of protein sequence	(153)
L117Cfs	Homo	AI, EC, NC	Nonfunctional	(157, 158)
R136X	Homo	AI, NC	Interfere with Fam20A–Fam20C dimer/tetramer formation, removes 75% of protein sequence	(125, 153, 159, 160)
L173R	Homo	AI, NC	Impaired folding, L173 participates in hydrophobic interactions	(153)
D197_I214delinsV	CH	AI	Fam20C interface, disulfide disruption, reduced secretion and activity	(124)
L205Cfs	CH	AI	Hypomorphic, alters 62% of protein sequence	(127, 153)
I214Nfs	CH	AI, NC	Destabilization, interferes with Fam20A–Fam20C dimer/tetramer formation, alters 60% of protein sequence	(153)
Q241-R271del	Homo	AI, NC	Interferes with Fam20A–Fam20C dimer/tetramer formation, destabilization	(159)
R243X	CH	AI, NC	Destabilization, R243 participates in polar contacts, removes 45% of protein sequence	(153)
E245Gfs	CH	AI, NC	Interferes with Fam20A–Fam20C dimer/tetramer formation, alters 55% of protein sequence	(155)
F252del	CH	AI, NC	Interferes with Fam20A–Fam20C dimer/tetramer formation	(153)
Y253C	CH	AI	Destabilization, Y253 participates in polar contacts, interferes with Fam20A–Fam20C dimer/tetramer formation	(127)
R271Sfs	Homo	AI	Destabilization, alters 50% of protein sequence	(124)
R276X	CH	AI	Loss of “kinase” domain, removes 49% of protein sequence	(124)
S303Cfs	Homo	AI, NC	Destabilization, alters 44% of protein sequence	(153)
F305Lfs	CH; Homo	AI, NC	Interferes with Fam20A–Fam20C dimer/tetramer formation	(153, 157, 158, 161)
E326Gfs	CH	AI	Destabilization, alters 40% of protein sequence	(157)
C330R	CH	AI	Disruption of disulfide	(162)
G331D	Homo	AI	Destabilization, introduces steric clash	(159)
R361C	CH	AI	Destabilization, R361 participates in polar contacts, interferes with Fam20A–Fam20C dimer/tetramer formation	(127)
R392Pfs	Homo	AI, NC	Destabilizing, alters 28% of protein sequence	(124, 163)
D403N	CH	AI	Impaired folding, disrupts multiple polar contacts	(72)
D410Pfs	CH	AI	Destabilization, D410 participates in polar contacts, alters 24% of protein sequence	(153)
F417Vfs	CH	AI	Destabilization, alters 23% of protein sequence	(127)
A432T	CH	AI	Destabilization, larger side chain introduces steric clashes	(162)
G436E	Homo	AI	Interferes with salt bridges	(157)
S450Pfs	Homo	AI, NC	Destabilization, alters 17% of protein sequence	(153)
K457X	Homo	AI, NC	Destabilization, removes 16% of protein sequence	(153)
R478X	CH; Homo	AI, NC	Destabilization, R478 participates in polar contacts, removes 11% of protein sequence	(153, 159)
E483Kfs	Homo	AI, NC, EC	Destabilization, alters 11% of protein sequence	(164)
H494fs	CH	AI, NC, EC	Destabilizing, alters 9% of protein sequence	(154)
L495Tfs	Homo	AI, NC, EC	Destabilizing, alters 9% of protein sequence	(154)
L495Nfs	Homo	AI, NC	Destabilizing, alters 9% of protein sequence	(153)
I505Sfs	Homo	AI, NC	Destabilizing, I505 participates in multiple hydrophobic interactions, alters 6% of protein sequence.	(153)
T508Kfs	Homo	AI	Destabilizing, T508 participates in polar contacts, alters 6% of protein sequence	(165)
54.7kb duplication	CH	AI	Unknown.	(162)

AI, Amelogenesis imperfecta; CH, Compound heterozygous; del, deletion; delins, deletion and insertion; EC, Ectopic calcification; fs, Frameshift; Het, Heterozygous; Homo, Homozygous; NC, nephrocalcinosis; X, STOP/Termination.

Clinical presentation is heterogeneous and the classifications presented here reflect the symptomology reported in the literature.

The roles of the Fam20 kinases in disease transcend our current knowledge, which is evident from preliminary studies pointing to potential roles of Fam20C in diseases beyond biomineralization and cardiac function (36, 128, 129, 130). Developing inhibitors/activators for Fam20B or C makes sense at this point due to their usefulness as academic tools. To date, only one inhibitor, FL-1607, has been developed for Fam20C, and no proper *in vitro* target engagement or biochemical binding/inhibitory assays have been shown for this compound (71). It is expected that *in vivo* targeting of Fam20 kinases would elicit major side effects owing to the diverse substrates essential for organism function (11, 56). The following section provides the evolutionary perspective of the Fam20 family from early invertebrates to mammals.

## Fam20 and animal evolution

Fam20 orthologues are observed across the animal kingdom from sponge to mammals and early invertebrates have a single copy of the Fam20 gene (Fig. 1) (29). *Amphimedon queenslandica* or sponge is considered to be the oldest animal phylum (131) and exhibits a single copy of the Fam20 gene, which has Fam20B-like glycan kinase activity and produces phosphorylated xylose residues on tetrasaccharide linkers (29). Cnidarians such as *Hydra magnipapillata* also exhibit a single Fam20B-like protein (29), which robustly phosphorylates xylose residues and is thought to contribute to CS peptidoglycan chain extension, a function conserved through to mammals (30). An interesting exception is the nematode *Caenorhabditis elegans* (*C. elegans*) as it is, to date, the only organism known that does not have a Fam20B-like kinase activity (51). Even though proteoglycan biosynthesis in *C. elegans* is remarkably conserved when compared with that in humans, only unphosphorylated xylose is detected in the tetrasaccharide linker of *C. elegans* CS proteogylcans (132), highlighting the absence of Fam20B activity (51). Instead, Fam20 in *C. elegans* (known as FAMK-1) is a protein kinase with the same SxE substrate preference as mammalian Fam20C (51, 133). A study of FAMK-1 in *C. elegans* to uncover its ancestral roles revealed that it is involved in many physiological processes contributing to fertility, embryogenesis, and development (133). During embryogenesis, FAMK-1 prevents multinucleation, which can be overcome by elevating the temperature or lowering cortical stiffness (133). In adults, FAMK-1 expression in the spermatheca, a tissue that undergoes repeated mechanical strain controlled by calcium fluxes, is important for fertility (133). In the context of the organism, it is clear that Fam20C activity is required in the late secretory pathway or outside the cell for function (133).

The advent of two members in Fam20 family is first observed in arthropods (29). *Drosophila melanogaster* has one copy each of Fam20B and Fam20C (29). In fact, Fam20C phosphorylates *Drosophila* egg yolk proteins *in vitro* (5), which are the closest functional analogs of vitellogenin and phosvitin (134). As stated previously, phosvitin, one of the most heavily phosphorylated proteins known, is a Fam20C substrate (5). Phosvitin is cleaved from vitellogenin, the major egg yolk protein found in all egg-laying animals (4), and largely consists of long stretches of serine residues that are phosphorylated by Fam20C despite the absence of glutamate residues (5). Phosphorylation of vitellogenin and/or its phosvitin domains occurs in birds, fish, worm, and insect yolk proteins (5), making this a widespread and evolutionarily conserved modification. It is duly noted that the functional consequences of these phosphorylation events have yet to be determined. Fam20C also plays important roles in *Apis* sp. or the honeybee where it phosphorylates royal jelly proteins (135). An in-depth phosphoproteomics study of royal jelly proteins determined that they are phosphorylated mainly on SxE sites likely by a Fam20C-like protein in the hypopharyngeal and mandibular glands of nurse bees from where royal jelly is secreted (135). Royal jelly is an indispensable dietary component of the queen bee and possesses antibacterial, anticancer, antihypertensive, and antioxidative effects that coincidentally benefit human health (135, 136, 137). Significantly, the antimicrobial activities of royal jelly are influenced by phosphorylation in complex ways (135).

While the role of Fam20C in biomineralization in vertebrates is well documented, the study of Fam20’s role in invertebrate biomineralization is in its infancy. A recent study characterized Fam20 cDNA from the pearl oyster, *Pinctada fucata*, and determined that it was expressed in the mantle edge positioned to play a role in shell formation (138). Furthermore, its expression increases in the stage of development when the shell is first forming and knockdown of Fam20 *in vivo* by RNA interference resulted in the formation of abnormal calcium carbonate crystals during shell formation (138). It is intriguing that Fam20C could be involved in calcium carbonate as well as calcium phosphate biomineralization processes, nevertheless it remains to be shown that *P. fucata* Fam20 displays Fam20C kinase activity on relevant substrates. Echinoderms such as *Strongylocentrotus purpuratus* or sea urchins exhibit a duplication of Fam20C wherein it has one copy of Fam20B and 2 copies of the Fam20C genes (29). Protochordates such as *Branchiostoma* and *Saccoglossus* exhibit both Fam20B and Fam20C; however, the tunicates such as *Ciona intestinalis* and *Oikopleura dioica* seem to have a single Fam20 gene exhibiting Fam20B-like functions (29). The reason is unclear; however, incomplete genome sequencing could be a contributing factor for this “absence” (29).

Fam20A is first observed in fish (139). In fact, fish express three copies of Fam20C and one copy each of Fam20B and Fam20A (29). Vertebrates have all three members of this subfamily, Fam20A, B, and C, while invertebrates/protochordates do not possess a Fam20A orthologue. This may be attributable to the need for enhanced Fam20C activity, which presumably would promote biomineralization necessary for the formation of bones and tooth enamel. It is a mystery why divergent animal species have maintained different Fam20 protein activities, but as pointed to previously, these phylogenetic analyses demonstrate that the Fam20B glycan kinase is likely the ancestral kinase (29). Fam20A may have been derived from Fam20C, lost its kinase activity but gained the function of activating Fam20C as a pseudokinase partner in vertebrates (29).

## Concluding remarks

Since 1883, secreted proteins have been known to be phosphorylated. The identification of Fam20C in 2012 displaced the intracellular CKs as genuine casein kinases and opened up a new field wherein over 100 substrates across the phosphoproteome were linked to a single secreted atypical kinase (11, 56). With a preferred motif of SxE/pS, Fam20C can account for approximately two-thirds of the secreted phosphoproteome. But, a large fraction of secretory phosphoproteins exhibits pThr, non-SxE pSer, and pTyr phosphorylation events, which may not be attributable to Fam20C. The field of secretory pathway and extracellular phosphorylation is poised to expand rapidly with the continued characterization of the kinases that function in these environments. Most of the secretory pathway kinases’ activities and functions have yet to be elucidated. It is unknown whether the other FJX and VLK family proteins are kinases and if so whether their substrates are proteins, lipids, or metabolites. On the other hand, we have made significant progress with the subfamily of secreted kinases composed of actual kinases, Fam20B and C and the pseudokinase Fam20A (Fig. 5). With established links to human skeletal diseases, the initial roles of the Fam20 family were thought to be focused on biomineralization; however, identification of SxE/pS motifs in over two-thirds of all secreted phosphoproteome including plasma, serum, cerebrospinal fluid, neuropeptides, and extracellular matrix components points to a diverse function of Fam20C. Indeed, our work with the heart-specific Fam20C knockout mouse revealed the quintessential role of Fam20C in maintaining cardiac health (101, 102). Furthermore, roles of Fam20B and Fam20C in invertebrate organisms suggest roles in glycan function, mollusk shell formation, insect egg development, beehive nutrition, and fertility of nematodes. Other groups have also reported diverse substrates for Fam20C playing essential roles in endoplasmic reticulum homeostasis, coagulation, nutrition, and hormonal regulations. Thus, organ-specific focus on Fam20C should reveal further systematic functions of Fam20C modulating a diverse set of substrates. Indeed, activators of Fam20C may benefit nonlethal Raine patients as well as protect against heart disease and other potential systemic health issues. Thus, the roles of the Fam20 family extend far beyond biomineralization, and greater focus should be put on identifying these multiple roles in diverse systems. We believe that the Fam20 family is just the tip of the iceberg since multiple secretory pathway kinases remain enigmatic. In fact, recent studies have revealed that the kinome possibly expands far beyond the 540 kinases with predicted kinases and pseudokinases exhibiting diverse functions beyond phosphate transfer (119, 120). Identification of the Fam20 family is a testament to the fact that atypical kinases exhibit catalytic residues, structural features, and cellular localizations outside of conventional knowledge. Hence, we have just scratched the surface of the physiological significance of extracellular phosphorylation and many exciting prospects await the field for the near future.

## Conflict of interest

The authors declare no conflicts of interest with the contents of this article.

## References

[1] Hammarsten O. (1883). Zur Frage ob das Caseïn ein einheitlicher Stoff sei. Z. Physiol. Chem..

[2] Leven P.A., Alsberg C. (1900). Zur chemie der paranucleinsaure. Hopper-Seyler’s Z. Physiol. Chem..

[3] Meggio F., Boulton A.P., Marchiori F., Borin G., Lennon D.P., Calderan A., Pinna L.A. (1988). Substrate-specificity determinants for a membrane-bound casein kinase of lactating mammary gland. A study with synthetic peptides. Eur. J. Biochem..

[4] Byrne B.M., van het Schip A.D., van de Klundert J.A., Arnberg A.C., Gruber M., Ab G. (1984). Amino acid sequence of phosvitin derived from the nucleotide sequence of part of the chicken vitellogenin gene. Biochemistry.

[5] Cozza G., Moro E., Black M., Marin O., Salvi M., Venerando A., Tagliabracci V.S., Pinna L.A. (2018). The Golgi 'casein kinase' Fam20C is a genuine 'phosvitin kinase' and phosphorylates polyserine stretches devoid of the canonical consensus. FEBS J..

[6] Burnett G., Kennedy E.P. (1954). The enzymatic phosphorylation of proteins. J. Biol. Chem..

[7] Rabinowitz M., Lipmann F. (1960). Reversible phosphate transfer between yolk phosphoprotein and adenosine triphosphate. J. Biol. Chem..

[8] Rodnight R., Lavin B.E. (1964). Phosvitin kinase from brain: Activation by ions and subcellular distribution. Biochem. J..

[9] Manning G., Whyte D.B., Martinez R., Hunter T., Sudarsanam S. (2002). The protein kinase complement of the human genome. Science.

[10] Ishikawa H.O., Xu A., Ogura E., Manning G., Irvine K.D. (2012). The Raine syndrome protein FAM20C is a Golgi kinase that phosphorylates bio-mineralization proteins. PLoS One.

[11] Tagliabracci V.S., Engel J.L., Wen J., Wiley S.E., Worby C.A., Kinch L.N., Xiao J., Grishin N.V., Dixon J.E. (2012). Secreted kinase phosphorylates extracellular proteins that regulate biomineralization. Science.

[12] Ishikawa H.O., Takeuchi H., Haltiwanger R.S., Irvine K.D. (2008). Four-jointed is a Golgi kinase that phosphorylates a subset of cadherin domains. Science.

[13] Cui J., Xiao J., Tagliabracci V.S., Wen J., Rahdar M., Dixon J.E. (2015). A secretory kinase complex regulates extracellular protein phosphorylation. Elife.

[14] Wen J., Xiao J., Rahdar M., Choudhury B.P., Cui J., Taylor G.S., Esko J.D., Dixon J.E. (2014). Xylose phosphorylation functions as a molecular switch to regulate proteoglycan biosynthesis. Proc. Natl. Acad. Sci. U. S. A..

[15] Koike T., Izumikawa T., Tamura J., Kitagawa H. (2009). FAM20B is a kinase that phosphorylates xylose in the glycosaminoglycan-protein linkage region. Biochem. J..

[16] Tagliabracci V.S., Xiao J., Dixon J.E. (2013). Phosphorylation of substrates destined for secretion by the Fam20 kinases. Biochem. Soc. Trans..

[17] Zhu Q., Venzke D., Walimbe A.S., Anderson M.E., Fu Q., Kinch L.N., Wang W., Chen X., Grishin N.V., Huang N., Yu L., Dixon J.E., Campbell K.P., Xiao J. (2016). Structure of protein O-mannose kinase reveals a unique active site architecture. Elife.

[18] Yoshida-Moriguchi T., Willer T., Anderson M.E., Venzke D., Whyte T., Muntoni F., Lee H., Nelson S.F., Yu L., Campbell K.P. (2013). SGK196 is a glycosylation-specific O-mannose kinase required for dystroglycan function. Science.

[19] Bordoli M.R., Yum J., Breitkopf S.B., Thon J.N., Italiano J.E., Xiao J., Worby C., Wong S.K., Lin G., Edenius M., Keller T.L., Asara J.M., Dixon J.E., Yeo C.Y., Whitman M. (2014). A secreted tyrosine kinase acts in the extracellular environment. Cell.

[20] Walimbe A.S., Okuma H., Joseph S., Yang T., Yonekawa T., Hord J.M., Venzke D., Anderson M.E., Torelli S., Manzur A., Devereaux M., Cuellar M., Prouty S., Ocampo Landa S., Yu L. (2020). POMK regulates dystroglycan function via LARGE1-mediated elongation of matriglycan. Elife.

[21] Hareza A., Bakun M., Świderska B., Dudkiewicz M., Koscielny A., Bajur A., Jaworski J., Dadlez M., Pawłowski K. (2018). Phosphoproteomic insights into processes influenced by the kinase-like protein DIA1/C3orf58. PeerJ.

[22] Dudkiewicz M., Lenart A., Pawłowski K. (2013). A novel predicted calcium-regulated kinase family implicated in neurological disorders. PLoS One.

[23] Tennant-Eyles A.J., Moffitt H., Whitehouse C.A., Roberts R.G. (2011). Characterisation of the FAM69 family of cysteine-rich endoplasmic reticulum proteins. Biochem. Biophys. Res. Commun..

[24] Probst B., Rock R., Gessler M., Vortkamp A., Püschel A.W. (2007). The rodent four-jointed ortholog Fjx1 regulates dendrite extension. Dev. Biol..

[25] Chai S.J., Ahmad Zabidi M.M., Gan S.P., Rajadurai P., Lim P.V.H., Ng C.C., Yap L.F., Teo S.H., Lim K.P., Patel V., Cheong S.C. (2019). An oncogenic role for four-jointed box 1 (FJX1) in nasopharyngeal carcinoma. Dis. Markers.

[26] Chai S.J., Yap Y.Y., Foo Y.C., Yap L.F., Ponniah S., Teo S.H., Cheong S.C., Patel V., Lim K.P. (2015). Identification of four-jointed box 1 (FJX1)-specific peptides for immunotherapy of nasopharyngeal carcinoma. PLoS One.

[27] Hsu C.Y., Chang G.C., Chen Y.J., Hsu Y.C., Hsiao Y.J., Su K.Y., Chen H.Y., Lin C.Y., Chen J.S., Chen Y.J., Hong Q.S., Ku W.H., Wu C.Y., Ho B.C., Chiang C.C. (2018). FAM198B is associated with prolonged survival and inhibits metastasis in lung adenocarcinoma via blockage of ERK-mediated MMP-1 expression. Clin. Cancer Res..

[28] Wei Z., Liu T., Lei J., Wu Y., Wang S., Liao K. (2018). Fam198a, a member of secreted kinase, secrets through caveolae biogenesis pathway. Acta Biochim. Biophys. Sin. (Shanghai).

[29] Zhang H., Zhu Q., Cui J., Wang Y., Chen M.J., Guo X., Tagliabracci V.S., Dixon J.E., Xiao J. (2018). Structure and evolution of the Fam20 kinases. Nat. Commun..

[30] Yamada S., Morimoto H., Fujisawa T., Sugahara K. (2007). Glycosaminoglycans in Hydra magnipapillata (Hydrozoa, Cnidaria): Demonstration of chondroitin in the developing nematocyst, the sting organelle, and structural characterization of glycosaminoglycans. Glycobiology.

[31] Nadanaka S., Zhou S., Kagiyama S., Shoji N., Sugahara K., Sugihara K., Asano M., Kitagawa H. (2013). EXTL2, a member of the EXT family of tumor suppressors, controls glycosaminoglycan biosynthesis in a xylose kinase-dependent manner. J. Biol. Chem..

[32] Vogel P., Hansen G.M., Read R.W., Vance R.B., Thiel M., Liu J., Wronski T.J., Smith D.D., Jeter-Jones S., Brommage R. (2012). Amelogenesis imperfecta and other biomineralization defects in Fam20a and Fam20c null mice. Vet. Pathol..

[33] Eames B.F., Yan Y.L., Swartz M.E., Levic D.S., Knapik E.W., Postlethwait J.H., Kimmel C.B. (2011). Mutations in fam20b and xylt1 reveal that cartilage matrix controls timing of endochondral ossification by inhibiting chondrocyte maturation. PLoS Genet..

[34] Tian Y., Ma P., Liu C., Yang X., Crawford D.M., Yan W., Bai D., Qin C., Wang X. (2015). Inactivation of Fam20B in the dental epithelium of mice leads to supernumerary incisors. Eur. J. Oral Sci..

[35] Wu J., Tian Y., Han L., Liu C., Sun T., Li L., Yu Y., Lamichhane B., D'Souza R.N., Millar S.E., Krumlauf R., Ornitz D.M., Feng J.Q., Klein O., Zhao H. (2020). FAM20B-catalyzed glycosaminoglycans control murine tooth number by restricting FGFR2b signaling. BMC Biol..

[36] Ma P., Yan W., Tian Y., Wang J., Feng J.Q., Qin C., Cheng Y.S., Wang X. (2016). Inactivation of Fam20B in joint cartilage leads to chondrosarcoma and postnatal ossification defects. Sci. Rep..

[37] Liu X., Li N., Zhang H., Liu J., Zhou N., Ran C., Chen X., Lu Y., Wang X., Qin C., Xiao J., Liu C. (2018). Inactivation of Fam20b in the neural crest-derived mesenchyme of mouse causes multiple craniofacial defects. Eur. J. Oral Sci..

[38] Kuroda Y., Murakami H., Enomoto Y., Tsurusaki Y., Takahashi K., Mitsuzuka K., Ishimoto H., Nishimura G., Kurosawa K. (2019). A novel gene (FAM20B encoding glycosaminoglycan xylosylkinase) for neonatal short limb dysplasia resembling Desbuquois dysplasia. Clin. Genet..

[39] Lei J., Deng H., Ran Y., Lv Y., Amhare A.F., Wang L., Guo X., Han J., Lammi M.J. (2020). Altered expression of aggrecan, FAM20B, B3GALT6, and EXTL2 in patients with osteoarthritis and Kashin-beck disease. Cartilage.

[40] Lipmann F. (1933). Über die Bindung der Phosphorsäure in Phosphorproteinen. I. Biochem. Z..

[41] Mercier J.C. (1981). Phosphorylation of caseins, present evidence for an amino-acid triplet code post-translationally recognized by specific kinases. Biochimie.

[42] Mercier J.C., Grosclaude F., Ribadeau-Dumas B. (1971). Primary structure of bovine s1 casein. Complete sequence. Eur. J. Biochem..

[43] Bingham E.W., Farrell H.M., Basch J.J. (1972). Phosphorylation of casein. Role of the Golgi apparatus. J. Biol. Chem..

[44] Cozza G., Tagliabracci V.S., Dixon J.E., Pinna L.A. (2015). “Genuine” casein kinase (Fam20C): The mother of the phosphosecretome. Kinomics Chap 2,.

[45] Moore A., Boulton A.P., Heid H.W., Jarasch E.D., Craig R.K. (1985). Purification and tissue-specific expression of casein kinase from the lactating Guinea-pig mammary gland. Eur. J. Biochem..

[46] Duncan J.S., Wilkinson M.C., Burgoyne R.D. (2000). Purification of Golgi casein kinase from bovine milk. Biochem. J..

[47] Lasa M., Marin O., Pinna L.A. (1997). Rat liver Golgi apparatus contains a protein kinase similar to the casein kinase of lactating mammary gland. Eur. J. Biochem..

[48] Brunati A.M., Marin O., Bisinella A., Salviati A., Pinna L.A. (2000). Novel consensus sequence for the Golgi apparatus casein kinase, revealed using proline-rich protein-1 (PRP1)-derived peptide substrates. Biochem. J..

[49] Tibaldi E., Arrigoni G., Brunati A.M., James P., Pinna L.A. (2006). Analysis of a sub-proteome which co-purifies with and is phosphorylated by the Golgi casein kinase. Cell Mol. Life Sci..

[50] Cozza G., Salvi M., Banerjee S., Tibaldi E., Tagliabracci V.S., Dixon J.E., Pinna L.A. (2015). A new role for sphingosine: Up-regulation of Fam20C, the genuine casein kinase that phosphorylates secreted proteins. Biochim. Biophys. Acta.

[51] Xiao J., Tagliabracci V.S., Wen J., Kim S.-A., Dixon J.E. (2013). Crystal structure of the Golgi casein kinase. Proc. Natl. Acad. Sci. U. S. A..

[52] Carrascal M., Gay M., Ovelleiro D., Casas V., Gelpí E., Abian J. (2010). Characterization of the human plasma phosphoproteome using linear ion trap mass spectrometry and multiple search engines. J. Proteome Res..

[53] Bahl J.M., Jensen S.S., Larsen M.R., Heegaard N.H. (2008). Characterization of the human cerebrospinal fluid phosphoproteome by titanium dioxide affinity chromatography and mass spectrometry. Anal. Chem..

[54] Zhou W., Ross M.M., Tessitore A., Ornstein D., Vanmeter A., Liotta L.A., Petricoin E.F. (2009). An initial characterization of the serum phosphoproteome. J. Proteome Res..

[55] Lietz C.B., Toneff T., Mosier C., Podvin S., O'Donoghue A.J., Hook V. (2018). Phosphopeptidomics reveals differential phosphorylation states and novel SxE phosphosite motifs of neuropeptides in dense core secretory vesicles. J. Am. Soc. Mass Spectrom..

[56] Tagliabracci V.S., Wiley S.E., Guo X., Kinch L.N., Durrant E., Wen J., Xiao J., Cui J., Nguyen K.B., Engel J.L., Coon J.J., Grishin N., Pinna L.A., Pagliarini D.J., Dixon J.E. (2015). A single kinase generates the majority of the secreted phosphoproteome. Cell.

[57] Ramos-Molina B., Lindberg I. (2015). Phosphorylation and alternative splicing of 7B2 reduce prohormone convertase 2 activation. Mol. Endocrinol..

[58] Cozza G., Salvi M., Tagliabracci V.S., Pinna L.A. (2017). Fam20C is under the control of sphingolipid signaling in human cell lines. FEBS J..

[59] Fang Z.H., Visker M., Miranda G., Delacroix-Buchet A., Bovenhuis H., Martin P. (2016). The relationships among bovine αS-casein phosphorylation isoforms suggest different phosphorylation pathways. J. Dairy Sci..

[60] Anema S.G., de Kruif C.G. (2013). Protein composition of different sized casein micelles in milk after the binding of lactoferrin or lysozyme. J. Agric. Food Chem..

[61] Holt C., Carver J.A., Ecroyd H., Thorn D.C. (2013). Invited review: Caseins and the casein micelle: Their biological functions, structures, and behavior in foods. J. Dairy Sci..

[62] Oya K., Ishida K., Nishida T., Sato S., Kishino M., Hirose K., Ogawa Y., Ikebe K., Takeshige F., Yasuda H., Komori T., Toyosawa S. (2017). Immunohistochemical analysis of dentin matrix protein 1 (Dmp1) phosphorylation by Fam20C in bone: Implications for the induction of biomineralization. Histochem. Cell Biol..

[63] Kinoshita Y., Hori M., Taguchi M., Fukumoto S. (2014). Functional analysis of mutant FAM20C in Raine syndrome with FGF23-related hypophosphatemia. Bone.

[64] Tibaldi E., Brocca A., Sticca A., Gola E., Pizzi M., Bordin L., Pagano M.A., Mazzorana M., Donà G., Violi P., Marin O., Romano A., Angeli P., Carraro A., Brunati A.M. (2020). Fam20C-mediated phosphorylation of osteopontin is critical for its secretion but dispensable for its action as a cytokine in the activation of hepatic stellate cells in liver fibrogenesis. FASEB J..

[65] Schytte G.N., Christensen B., Bregenov I., Kjøge K., Scavenius C., Petersen S.V., Enghild J.J., Sørensen E.S. (2020). FAM20C phosphorylation of the RGDSVVYGLR motif in osteopontin inhibits interaction with the αvβ3 integrin. J. Cell Biochem..

[66] Du E.X., Wang X.F., Yang W.C., Kaback D., Yee S.P., Qin C.L., George A., Hao J.J. (2015). Characterization of Fam20C expression in odontogenesis and osteogenesis using transgenic mice. Int. J. Oral Sci..

[67] Liu C., Zhang H., Jani P., Wang X., Lu Y., Li N., Xiao J., Qin C. (2018). FAM20C regulates osteoblast behaviors and intracellular signaling pathways in a cell-autonomous manner. J. Cell Physiol..

[68] Liu C., Zhou N., Wang Y., Zhang H., Jani P., Wang X., Lu Y., Li N., Xiao J., Qin C. (2018). Abrogation of Fam20c altered cell behaviors and BMP signaling of immortalized dental mesenchymal cells. Exp. Cell Res..

[69] Liu P., Zhang H., Liu C., Wang X., Chen L., Qin C. (2014). Inactivation of Fam20C in cells expressing type I collagen causes periodontal disease in mice. PLoS One.

[70] Ma P., Yan W., Tian Y., He J., Brookes S.J., Wang X. (2016). The importance of serine phosphorylation of ameloblastin on enamel formation. J. Dent. Res..

[71] Qin Z., Wang P., Li X., Zhang S., Tian M., Dai Y., Fu L. (2016). Systematic network-based discovery of a Fam20C inhibitor (FL-1607) with apoptosis modulation in triple-negative breast cancer. Mol. Biosyst..

[72] Wang S.K., Reid B.M., Dugan S.L., Roggenbuck J.A., Read L., Aref P., Taheri A.P., Yeganeh M.Z., Simmer J.P., Hu J.C. (2014). FAM20A mutations associated with enamel renal syndrome. J. Dent. Res..

[73] Wang S.K., Samann A.C., Hu J.C., Simmer J.P. (2013). FAM20C functions intracellularly within both ameloblasts and odontoblasts *in vivo*. J. Bone Miner. Res..

[74] Wang X., Hao J., Xie Y., Sun Y., Hernandez B., Yamoah A.K., Prasad M., Zhu Q., Feng J.Q., Qin C. (2010). Expression of FAM20C in the osteogenesis and odontogenesis of mouse. J. Histochem. Cytochem..

[75] Wang X., Jung J., Liu Y., Yuan B., Lu Y., Feng J.Q., Qin C. (2013). The specific role of FAM20C in amelogenesis. J. Dent. Res..

[76] Wang X., Wang S., Li C., Gao T., Liu Y., Rangiani A., Sun Y., Hao J., George A., Lu Y., Groppe J., Yuan B., Feng J.Q., Qin C. (2012). Inactivation of a novel FGF23 regulator, FAM20C, leads to hypophosphatemic rickets in mice. PLoS Genet..

[77] Wang X., Wang S., Lu Y., Gibson M.P., Liu Y., Yuan B., Feng J.Q., Qin C. (2012). FAM20C plays an essential role in the formation of murine teeth. J. Biol. Chem..

[78] Yan W.J., Ma P., Tian Y., Wang J.Y., Qin C.L., Feng J.Q., Wang X.F. (2017). The importance of a potential phosphorylation site in enamelin on enamel formation. Int. J. Oral Sci..

[79] Kang T., Boland B.B., Alarcon C., Grimsby J.S., Rhodes C.J., Larsen M.R. (2019). Proteomic analysis of restored insulin production and trafficking in obese diabetic mouse pancreatic islets following euglycemia. J. Proteome Res..

[80] Zhang J., Zhu Q., Wang X., Yu J., Chen X., Wang J., Wang X., Xiao J., Wang C.C., Wang L. (2018). Secretory kinase Fam20C tunes endoplasmic reticulum redox state via phosphorylation of Ero1α. EMBO J..

[81] Yu J., Li T., Liu Y., Wang X., Zhang J., Wang X., Shi G., Lou J., Wang L., Wang C.C., Wang L. (2020). Phosphorylation switches protein disulfide isomerase activity to maintain proteostasis and attenuate ER stress. EMBO J..

[82] Hatahet F., Ruddock L.W. (2009). Protein disulfide isomerase: A critical evaluation of its function in disulfide bond formation. Antioxid. Redox Signal..

[83] Wang L., Wang X., Wang C.C. (2015). Protein disulfide-isomerase, a folding catalyst and a redox-regulated chaperone. Free Radic. Biol. Med..

[84] Hecht T.K., Blank B., Steger M., Lopez V., Beck G., Ramazanov B., Mann M., Tagliabracci V., von Blume J. (2020). Fam20C regulates protein secretion by Cab45 phosphorylation. J. Cell Biol..

[85] Tagliabracci V.S., Engel J.L., Wiley S.E., Xiao J., Gonzalez D.J., Appaiah H.N., Koller A., Nizet V., White K.E., Dixon J.E. (2014). Dynamic regulation of FGF23 by Fam20C phosphorylation, GalNAc-T3 glycosylation, and furin proteolysis. Proc. Natl. Acad. Sci. U. S. A..

[86] Blombaeck B., Blombaeck M., Edman P., Hessel B. (1962). Amino-acid sequence and the occurrence of phosphorus in human fibrinopeptides. Nature.

[87] de Maat M. (1995). Regulation and Modulation of the Plasma Fibrinogen Level.

[88] Hanna L.S., Scheraga H.A., Francis C.W., Marder V.J. (1984). Comparison of structures of various human fibrinogens and a derivative thereof by a study of the kinetics of release of fibrinopeptides. Biochemistry.

[89] Regañón E., Vila V., Aznar J., Laiz B. (1989). Human fibrinogen heterogeneity. A study of limited fibrinogen degradation. Clin. Chim. Acta.

[90] Da Q., Han H., Valladolid C., Fernández M., Khatlani T., Pradhan S., Nolasco J., Matsunami R.K., Engler D.A., Cruz M.A., Vijayan K.V. (2019). *In vitro* phosphorylation of von Willebrand factor by FAM20c enhances its ability to support platelet adhesion. J. Thromb. Haemost..

[91] Qiu Y., Poppleton E., Mekkat A., Yu H., Banerjee S., Wiley S.E., Dixon J.E., Kaplan D.L., Lin Y.S., Brodsky B. (2018). Enzymatic phosphorylation of ser in a type I collagen peptide. Biophys. J..

[92] Nilsson Ekdahl K., Nilsson B. (1997). Phosphorylation of complement component C3 after synthesis in U937 cells by a putative protein kinase, casein kinase 2, which is regulated by CD11b: Evidence that membrane-bound proteases preferentially cleave phosphorylated C3. Biochem. J..

[93] Bhattacharyya N., Chong W.H., Gafni R.I., Collins M.T. (2012). Fibroblast growth factor 23: State of the field and future directions. Trends Endocrinol. Metab..

[94] Zhang H., Li L., Kesterke M.J., Lu Y., Qin C. (2019). High-phosphate diet improved the skeletal development of Fam20c-deficient mice. Cells Tissues Organs.

[95] Consortium A. (2000). Autosomal dominant hypophosphataemic rickets is associated with mutations in FGF23. Nat. Genet..

[96] Coresh J., Selvin E., Stevens L.A., Manzi J., Kusek J.W., Eggers P., Van Lente F., Levey A.S. (2007). Prevalence of chronic kidney disease in the United States. JAMA.

[97] Ben Djoudi Ouadda A., Gauthier M.S., Susan-Resiga D., Girard E., Essalmani R., Black M., Marcinkiewicz J., Forget D., Hamelin J., Evagelidis A., Ly K., Day R., Galarneau L., Corbin F., Coulombe B. (2019). Ser-phosphorylation of PCSK9 (proprotein convertase subtilisin-kexin 9) by Fam20C (family with sequence similarity 20, member C) kinase enhances its ability to degrade the LDLR (low-density lipoprotein receptor). Arterioscler. Thromb. Vasc. Biol..

[98] Ashraf Y., Duval S., Sachan V., Essalmani R., Susan-Resiga D., Roubtsova A., Hamelin J., Gerhardy S., Kirchhofer D., Tagliabracci V.S., Prat A., Kiss R.S., Seidah N.G. (2020). Proprotein convertase 7 (PCSK7) reduces apoA-V levels. FEBS J..

[99] Bers D.M. (2014). Cardiac sarcoplasmic reticulum calcium leak: Basis and roles in cardiac dysfunction. Annu. Rev. Physiol..

[100] Bers D.M. (2002). Cardiac excitation-contraction coupling. Nature.

[101] Pollak A.J., Haghighi K., Kunduri S., Arvanitis D.A., Bidwell P.A., Liu G.-S., Singh V.P., Gonzalez D.J., Sanoudou D., Wiley S.E., Dixon J.E., Kranias E.G. (2017). Phosphorylation of serine96 of histidine-rich calcium- binding protein by the Fam20C kinase functions to prevent cardiac arrhythmia. Proc. Natl. Acad. Sci. U. S. A..

[102] Pollak A.J., Liu C., Gudlur A., Mayfield J.E., Dalton N.D., Gu Y., Chen J., Heller Brown J., Hogan P.G., Wiley S.E., Peterson K.L., Dixon J.E. (2018). A secretory pathway kinase regulates sarcoplasmic reticulum Ca(2+) homeostasis and protects against heart failure. Elife.

[103] Arvanitis D.A., Vafiadaki E., Sanoudou D., Kranias E.G. (2011). Histidine-rich calcium binding protein: The new regulator of sarcoplasmic reticulum calcium cycling. J. Mol. Cell Cardiol..

[104] Harris E., Burki U., Marini-Bettolo C., Neri M., Scotton C., Hudson J., Bertoli M., Evangelista T., Vroling B., Polvikoski T., Roberts M., Töpf A., Bushby K., McArthur D., Lochmüller H. (2017). Complex phenotypes associated with STIM1 mutations in both coiled coil and EF-hand domains. Neuromuscul. Disord..

[105] Zipes D.P., Wellens H.J.J., Smeets J.L.R.M., Doevendans P.A., Josephson M.E., Kirchhof C., Vos M.A. (2000). Sudden cardiac death. Professor Hein J.J. Wellens: 33 Years of Cardiology and Arrhythmology.

[106] Park C.S., Chen S., Lee H., Cha H., Oh J.G., Hong S., Han P., Ginsburg K.S., Jin S., Park I., Singh V.P., Wang H.S., Franzini-Armstrong C., Park W.J., Bers D.M. (2013). Targeted ablation of the histidine-rich Ca(2+)-binding protein (HRC) gene is associated with abnormal SR Ca(2+)-cycling and severe pathology under pressure-overload stress. Basic Res. Cardiol..

[107] Simpson M.A., Hsu R., Keir L.S., Hao J., Sivapalan G., Ernst L.M., Zackai E.H., Al-Gazali L.I., Hulskamp G., Kingston H.M., Prescott T.E., Ion A., Patton M.A., Murday V., George A. (2007). Mutations in FAM20C are associated with lethal osteosclerotic bone dysplasia (Raine syndrome), highlighting a crucial molecule in bone development. Am. J. Hum. Genet..

[108] Simpson M.A., Scheuerle A., Hurst J., Patton M.A., Stewart H., Crosby A.H. (2009). Mutations in FAM20C also identified in non-lethal osteosclerotic bone dysplasia. Clin. Genet..

[109] Fradin M., Stoetzel C., Muller J., Koob M., Christmann D., Debry C., Kohler M., Isnard M., Astruc D., Desprez P., Zorres C., Flori E., Dollfus H., Doray B. (2011). Osteosclerotic bone dysplasia in siblings with a Fam20C mutation. Clin. Genet..

[110] Whyte M.P., McAlister W.H., Kim G.S., Sly W.S., Pierpont M.E., Brown D.M., Fallon M.D. (1985). Congenital sclerosing osteomalacia with cerebral calcification: A new, recessively inherited, syndrome which radiographically mimics carbonic anhydrase II deficiency. (Abstract). Am. J. Hum. Genet..

[111] Whyte M.P., McAlister W.H., Fallon M.D., Pierpont M.E., Bijanki V.N., Duan S., Otaify G.A., Sly W.S., Mumm S. (2017). Raine syndrome (OMIM #259775), caused by FAM20C mutation, is congenital sclerosing osteomalacia with cerebral calcification (OMIM 259660). J. Bone Miner. Res..

[112] Raine J., Winter R.M., Davey A., Tucker S.M. (1989). Unknown syndrome: Microcephaly, hypoplastic nose, exophthalmos, gum hyperplasia, cleft palate, low set ears, and osteosclerosis. J. Med. Genet..

[113] Rafaelsen S.H., Raeder H., Fagerheim A.K., Knappskog P., Carpenter T.O., Johansson S., Bjerknes R. (2013). Exome sequencing reveals FAM20c mutations associated with fibroblast growth factor 23-related hypophosphatemia, dental anomalies, and ectopic calcification. J. Bone Miner. Res..

[114] Sheth J., Bhavsar R., Gandhi A., Sheth F., Pancholi D. (2018). A case of Raine syndrome presenting with facial dysmorphy and review of literature. BMC Med. Genet..

[115] Hytönen M.K., Arumilli M., Lappalainen A.K., Owczarek-Lipska M., Jagannathan V., Hundi S., Salmela E., Venta P., Sarkiala E., Jokinen T., Gorgas D., Kere J., Nieminen P., Drögemüller C., Lohi H. (2016). Molecular characterization of three canine models of human rare bone diseases: Caffey, van den Ende-Gupta, and Raine syndromes. PLoS Genet..

[116] Acevedo A.C., Poulter J.A., Alves P.G., de Lima C.L., Castro L.C., Yamaguti P.M., Paula L.M., Parry D.A., Logan C.V., Smith C.E., Johnson C.A., Inglehearn C.F., Mighell A.J. (2015). Variability of systemic and oro-dental phenotype in two families with non-lethal Raine syndrome with FAM20C mutations. BMC Med. Genet..

[117] Eltan M., Alavanda C., Yavas Abali Z., Ergenekon P., Yalındag Ozturk N., Sakar M., Dagcinar A., Kirkgoz T., Kaygusuz S.B., Gokdemir Y., Elcioglu H.N., Guran T., Bereket A., Ata P., Turan S. (2020). A rare cause of hypophosphatemia: Raine syndrome changing clinical features with age. Calcif. Tissue Int..

[118] Boudeau J., Miranda-Saavedra D., Barton G.J., Alessi D.R. (2006). Emerging roles of pseudokinases. Trends Cell Biol..

[119] Black M.H., Osinski A., Gradowski M., Servage K.A., Pawłowski K., Tomchick D.R., Tagliabracci V.S. (2019). Bacterial pseudokinase catalyzes protein polyglutamylation to inhibit the SidE-family ubiquitin ligases. Science.

[120] Sreelatha A., Yee S.S., Lopez V.A., Park B.C., Kinch L.N., Pilch S., Servage K.A., Zhang J., Jiou J., Karasiewicz-Urbańska M., Łobocka M., Grishin N.V., Orth K., Kucharczyk R., Pawłowski K. (2018). Protein AMPylation by an evolutionarily conserved pseudokinase. Cell.

[121] Cui J., Zhu Q., Zhang H., Cianfrocco M.A., Leschziner A.E., Dixon J.E., Xiao J. (2017). Structure of Fam20A reveals a pseudokinase featuring a unique disulfide pattern and inverted ATP-binding. Elife.

[122] Murphy J.M., Zhang Q., Young S.N., Reese M.L., Bailey F.P., Eyers P.A., Ungureanu D., Hammaren H., Silvennoinen O., Varghese L.N., Chen K., Tripaydonis A., Jura N., Fukuda K., Qin J. (2014). A robust methodology to subclassify pseudokinases based on their nucleotide-binding properties. Biochem. J..

[123] Zeqiraj E., Filippi B.M., Deak M., Alessi D.R., van Aalten D.M. (2009). Structure of the LKB1-STRAD-MO25 complex reveals an allosteric mechanism of kinase activation. Science.

[124] Cho S.H., Seymen F., Lee K.E., Lee S.K., Kweon Y.S., Kim K.J., Jung S.E., Song S.J., Yildirim M., Bayram M., Tuna E.B., Gencay K., Kim J.W. (2012). Novel FAM20A mutations in hypoplastic amelogenesis imperfecta. Hum. Mutat..

[125] O'Sullivan J., Bitu C.C., Daly S.B., Urquhart J.E., Barron M.J., Bhaskar S.S., Martelli-Junior H., dos Santos Neto P.E., Mansilla M.A., Murray J.C., Coletta R.D., Black G.C., Dixon M.J. (2011). Whole-exome sequencing identifies FAM20A mutations as a cause of amelogenesis imperfecta and gingival hyperplasia syndrome. Am. J. Hum. Genet..

[126] Li L.L., Liu P.H., Xie X.H., Ma S., Liu C., Chen L., Qin C.L. (2016). Loss of epithelial FAM20A in mice causes amelogenesis imperfecta, tooth eruption delay and gingival overgrowth. Int. J. Oral Sci..

[127] Nitayavardhana I., Theerapanon T., Srichomthong C., Piwluang S., Wichadakul D., Porntaveetus T., Shotelersuk V. (2020). Four novel mutations of FAM20A in amelogenesis imperfecta type IG and review of literature for its genotype and phenotype spectra. Mol. Genet. Genomics.

[128] Trendowski M.R., Wheeler H.E., El-Charif O., Feldman D.R., Hamilton R.J., Vaughn D.J., Fung C., Kollmannsberger C., Einhorn L.H., Travis L.B., Dolan M.E. (2020). Clinical and genome-wide analysis of multiple severe cisplatin-induced neurotoxicities in adult-onset cancer survivors. Clin. Cancer Res..

[129] Du S., Guan S., Zhu C., Guo Q., Cao J., Guan G., Cheng W., Cheng P., Wu A. (2020). Secretory pathway kinase FAM20C, a marker for glioma invasion and malignancy, predicts poor prognosis of glioma. Oncotargets Ther..

[130] Kang J.U. (2013). Characterization of amplification patterns and target genes on the short arm of chromosome 7 in early-stage lung adenocarcinoma. Mol. Med. Rep..

[131] Pisani D., Pett W., Dohrmann M., Feuda R., Rota-Stabelli O., Philippe H., Lartillot N., Wörheide G. (2015). Genomic data do not support comb jellies as the sister group to all other animals. Proc. Natl. Acad. Sci. U. S. A..

[132] Yamada S., Okada Y., Ueno M., Iwata S., Deepa S.S., Nishimura S., Fujita M., Van Die I., Hirabayashi Y., Sugahara K. (2002). Determination of the glycosaminoglycan-protein linkage region oligosaccharide structures of proteoglycans from Drosophila melanogaster and Caenorhabditis elegans. J. Biol. Chem..

[133] Gerson-Gurwitz A., Worby C.A., Lee K.Y., Khaliullin R., Bouffard J., Cheerambathur D., Oegema K., Cram E.J., Dixon J.E., Desai A. (2019). Ancestral roles of the Fam20C family of secreted protein kinases revealed in C. elegans. J. Cell Biol..

[134] Terpstra P., Ab G. (1988). Homology of Drosophila yolk proteins and the triacylglycerol lipase family. J. Mol. Biol..

[135] Han B., Fang Y., Feng M., Lu X., Huo X., Meng L., Wu B., Li J. (2014). In-depth phosphoproteomic analysis of royal jelly derived from western and eastern honeybee species. J. Proteome Res..

[136] Bíliková K., Wu G., Šimúth J. (2001). Isolation of a peptide fraction from honeybee royal jelly as a potential antifoulbrood factor. Apidologie.

[137] Tamura T., Fujii A., Kuboyama N. (1987). [Antitumor effects of royal jelly (RJ)]. Nihon Yakurigaku Zasshi.

[138] Du J., Liu C., Xu G., Xie J., Xie L., Zhang R. (2018). Fam20C participates in the shell formation in the pearl oyster, Pinctada fucata. Sci. Rep..

[139] Nalbant D., Youn H., Nalbant S.I., Sharma S., Cobos E., Beale E.G., Du Y., Williams S.C. (2005). FAM20: An evolutionarily conserved family of secreted proteins expressed in hematopoietic cells. BMC Genomics.

[140] Hernández-Zavala A., Cortés-Camacho F., Palma Lara I., Godinez-Aguilar R., Espinosa-García A.M., Pérez-Durán J., Villanueva-Ocampo P., Ugarte-Briones C., Serrano-Bello C.A., Sanchez-Santiago P., Bonilla-Delgado J., Yañez-López M.A., Victoria-Acosta G., López-Ornelas A., García Alonso-Themann P. (2020). Two novel FAM20C variants in A family with Raine syndrome. Genes (Basel).

[141] Mameli C., Zichichi G., Mahmood N., Elalaoui S.C., Mirza A., Dharmaraj P., Burrone M., Cattaneo E., Sheth J., Gandhi A., Kochar G.S., Alkuraya F.S., Kabra M., Mercurio G., Zuccotti G. (2020). Natural history of non-lethal Raine syndrome during childhood. Orphanet J. Rare Dis..

[142] Elalaoui S.C., Al-Sheqaih N., Ratbi I., Urquhart J.E., O'Sullivan J., Bhaskar S., Williams S.S., Elalloussi M., Lyahyai J., Sbihi L., Cherkaoui Jaouad I., Sbihi A., Newman W.G., Sefiani A. (2016). Non lethal Raine syndrome and differential diagnosis. Eur. J. Med. Genet..

[143] Rolvien T., Kornak U., Schinke T., Amling M., Oheim R. (2019). A novel FAM20C mutation causing hypophosphatemic osteomalacia with osteosclerosis (mild Raine syndrome) in an elderly man with spontaneous osteonecrosis of the knee. Osteoporos. Int..

[144] Boissel S., Fallet-Bianco C., Chitayat D., Kremer V., Nassif C., Rypens F., Delrue M.-A., Dal Soglio D., Oligny L.L., Patey N., Flori E., Cloutier M., Dyment D., Campeau P., Karalis A. (2018). Genomic study of severe fetal anomalies and discovery of GREB1L mutations in renal agenesis. Genet. Med..

[145] Hung C.Y., Rodriguez M., Roberts A., Bauer M., Mihalek I., Bodamer O. (2019). A novel FAM20C mutation causes a rare form of neonatal lethal Raine syndrome. Am. J. Med. Genet. A.

[146] Mamedova E., Dimitrova D., Przhiyalkovskaya E., Buryakina S., Vasilyev E., Tiulpakov A., Belaya Z. (2019). Non-lethal raine syndrome in a middle-aged woman caused by a novel FAM20C mutation. Calcif. Tissue Int..

[147] Tamai K., Tada K., Takeuchi A., Nakamura M., Marunaka H., Washio Y., Tanaka H., Miya F., Okamoto N., Kageyama M. (2018). Fetal ultrasonographic findings including cerebral hyperechogenicity in a patient with non-lethal form of Raine syndrome. Am. J. Med. Genet. A.

[148] Takeyari S., Yamamoto T., Kinoshita Y., Fukumoto S., Glorieux F.H., Michigami T., Hasegawa K., Kitaoka T., Kubota T., Imanishi Y., Shimotsuji T., Ozono K. (2014). Hypophosphatemic osteomalacia and bone sclerosis caused by a novel homozygous mutation of the FAM20C gene in an elderly man with a mild variant of Raine syndrome. Bone.

[149] Seidahmed M.Z., Alazami A.M., Abdelbasit O.B., Al Hussein K., Miqdad A.M., Abu-Sa'da O., Mustafa T., Bahjat S., Alkuraya F.S. (2015). Report of a case of Raine syndrome and literature review. Am. J. Med. Genet. A.

[150] Kochar G.S., Choudhary A., Gadodia A., Gupta N., Simpson M.A., Crosby A.H., Kabra M. (2010). Raine syndrome: A clinical, radiographic and genetic investigation of a case from the Indian subcontinent. Clin. Dysmorphol..

[151] Ababneh F.K., AlSwaid A., Youssef T., Al Azzawi M., Crosby A., AlBalwi M.A. (2013). Hereditary deletion of the entire FAM20C gene in a patient with Raine syndrome. Am. J. Med. Genet. A.

[152] Cherkaoui Jaouad I., El Alloussi M., Chafai El Alaoui S., Laarabi F.Z., Lyahyai J., Sefiani A. (2015). Further evidence for causal FAM20A mutations and first case of amelogenesis imperfecta and gingival hyperplasia syndrome in Morocco: A case report. BMC Oral Health.

[153] Jaureguiberry G., De la Dure-Molla M., Parry D., Quentric M., Himmerkus N., Koike T., Poulter J., Klootwijk E., Robinette S.L., Howie A.J., Patel V., Figueres M.L., Stanescu H.C., Issler N., Nicholson J.K. (2012). Nephrocalcinosis (enamel renal syndrome) caused by autosomal recessive FAM20A mutations. Nephron Physiol..

[154] Kantaputra P.N., Bongkochwilawan C., Kaewgahya M., Ohazama A., Kayserili H., Erdem A.P., Aktoren O., Guven Y. (2014). Enamel-renal-gingival syndrome, hypodontia, and a novel FAM20A mutation. Am. J. Med. Genet. A.

[155] Wang Y.P., Lin H.Y., Zhong W.L., Simmer J.P., Wang S.K. (2019). Transcriptome analysis of gingival tissues of enamel-renal syndrome. J. Periodontal Res..

[156] Cabral R.M., Kurban M., Rothman L., Wajid M., Shimomura Y., Petukhova L., Christiano A.M. (2013). Autosomal recessive gingival hyperplasia and dental anomalies caused by a 29-base pair duplication in the FAM20A gene. J. Hum. Genet..

[157] Kantaputra P.N., Bongkochwilawan C., Lubinsky M., Pata S., Kaewgahya M., Tong H.J., Ketudat Cairns J.R., Guven Y., Chaisrisookumporn N. (2017). Periodontal disease and FAM20A mutations. J. Hum. Genet..

[158] Kantaputra P.N., Kaewgahya M., Khemaleelakul U., Dejkhamron P., Sutthimethakorn S., Thongboonkerd V., Iamaroon A. (2014). Enamel-renal-gingival syndrome and FAM20A mutations. Am. J. Med. Genet. A.

[159] Wang S.K., Aref P., Hu Y., Milkovich R.N., Simmer J.P., El-Khateeb M., Daggag H., Baqain Z.H., Hu J.C. (2013). FAM20A mutations can cause enamel-renal syndrome (ERS). PLoS Genet..

[160] Pego S.P.B., Coletta R.D., Dumitriu S., Iancu D., Albanyan S., Kleta R., Auricchio M.T., Santos L.A., Rocha B., Martelli-Junior H. (2017). Enamel-renal syndrome in 2 patients with a mutation in FAM20 A and atypical hypertrichosis and hearing loss phenotypes. Oral Surg. Oral Med. Oral Pathol. Oral Radiol..

[161] Hassib N.F., Shoeib M.A., ElSadek H.A., Wali M.E., Mostafa M.I., Abdel-Hamid M.S. (2020). Two new families with enamel renal syndrome: A novel FAM20A gene mutation and review of literature. Eur. J. Med. Genet..

[162] Poulter J.A., Smith C.E., Murrillo G., Silva S., Feather S., Howell M., Crinnion L., Bonthron D.T., Carr I.M., Watson C.M., Inglehearn C.F., Mighell A.J. (2015). A distinctive oral phenotype points to FAM20A mutations not identified by Sanger sequencing. Mol. Genet. Genomic Med..

[163] Koruyucu M., Seymen F., Gencay G., Gencay K., Tuna E.B., Shin T.J., Hyun H.K., Kim Y.J., Kim J.W. (2018). Nephrocalcinosis in amelogenesis imperfecta caused by the FAM20A mutation. Nephron.

[164] Dourado M.R., Dos Santos C.R.R., Dumitriu S., Iancu D., Albanyan S., Kleta R., Coletta R.D., Marques Mesquita A.T. (2019). Enamel renal syndrome: A novel homozygous FAM20A founder mutation in 5 new Brazilian families. Eur. J. Med. Genet..

[165] Volodarsky M., Zilberman U., Birk O.S. (2015). Novel FAM20A mutation causes autosomal recessive amelogenesis imperfecta. Arch. Oral Biol..

